# *Chlamydia trachomatis* from Australian Aboriginal people with trachoma are polyphyletic composed of multiple distinctive lineages

**DOI:** 10.1038/ncomms10688

**Published:** 2016-02-25

**Authors:** Patiyan Andersson, Simon R. Harris, Helena M. B. Seth Smith, James Hadfield, Colette O'Neill, Lesley T. Cutcliffe, Fiona P. Douglas, L. Valerie Asche, John D. Mathews, Susan I. Hutton, Derek S. Sarovich, Steven Y. C. Tong, Ian N. Clarke, Nicholas R. Thomson, Philip M. Giffard

**Affiliations:** 1 Global and Tropical Health Division, Menzies School of Health Research, Charles Darwin University, Darwin, Casuarina, Northern Territory 0811, Australia; 2 Pathogen Variation Programme, The Wellcome Trust Sanger Institute, Wellcome Trust Genome Campus, Cambridge CB10 1SA, UK; 3 Functional Genomics Centre Zürich, University of Zurich, Zurich CH-8057, Switzerland; 4 Institute for Veterinary Pathology, Vetsuisse Faculty, University of Zurich, Zurich CH-8057, Switzerland; 5 Department of Clinical and Experimental Science, Molecular Microbiology Group, University Medical School, Southampton General Hospital, Southampton SO16 6YD, UK; 6 School of Population and Global Health, University of Melbourne, Melbourne, Victoria 3010, Australia; 7 Department of Infectious and Tropical Diseases, London School of Hygiene and Tropical Medicine, London WC1E 7HT, UK; 8 School of Psychological and Clinical Sciences, Charles Darwin University, Casuarina, Northern Territory 0811, Australia

## Abstract

*Chlamydia trachomatis* causes sexually transmitted infections and the blinding disease trachoma. Current data on *C. trachomatis* phylogeny show that there is only a single trachoma-causing clade, which is distinct from the lineages causing urogenital tract (UGT) and lymphogranuloma venerum diseases. Here we report the whole-genome sequences of ocular *C. trachomatis* isolates obtained from young children with clinical signs of trachoma in a trachoma endemic region of northern Australia. The isolates form two lineages that fall outside the classical trachoma lineage, instead being placed within UGT clades of the *C. trachomatis* phylogenetic tree. The Australian trachoma isolates appear to be recombinants with UGT *C. trachomatis* genome backbones, in which loci that encode immunodominant surface proteins (*ompA* and *pmpEFGH*) have been replaced by those characteristic of classical ocular isolates. This suggests that ocular tropism and association with trachoma are functionally associated with some sequence variants of *ompA* and *pmpEFGH*.

## Abstract (web summary)

*Chlamydia trachomatis* isolates causing a blinding disease (trachoma) form a single lineage that is different from the lineages causing urogenital infections. Here, Andersson *et al*. show however that trachoma isolates from Australia are more closely related to urogenital strains than to other trachoma isolates.

## Graphical abstract

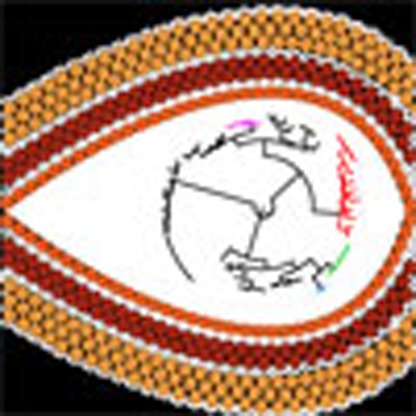

The genus *Chlamydia* encompasses bacteria that are obligate intracellular parasites with a distinctive life cycle. *Chlamydia trachomatis* is a human-specific pathogen that causes urogenital tract (UGT) and ocular infections^1^,^2^. Although essentially all *C. trachomatis* strains can cause conjunctivitis, trachoma is a chronic *C. trachomatis* ocular infection characterized by rounds of reinfection, hypersensitivity reactions with inflammation, conjunctival scarring and entropion, ultimately leading to corneal scarring, which may result in blindness^3^.

It is well established that strains of *C. trachomatis* differ as to whether they are primarily associated with UGT infections or trachoma. It has also long been known that different sequence variants of the *ompA* gene, encoding a major outer membrane protein, are markers for primary association with UGT infections or trachoma^4^,^5^,^6^,^7^. *OmpA* genotypes A, B, Ba and C are associated with trachoma, genotypes D-K associated with non-invasive UGT infections, and genotypes L1-L3 and L2b associated with invasive infections and lymphogranuloma venerum (LGV). Recent whole-genome sequence-based studies have shown that although recombination events mean that *ompA* sequence is not a reliable phylogenetic marker across the species, isolates with o*mpA* sequences that define the trachoma *ompA* genotypes all fall within a single clade of the *C. trachomatis* phylogenetic tree that shares a distant common ancestor with one of the two UGT lineages^8^,^9^,^10^. This ‘classical ocular' lineage does not include UGT or LGV *ompA* genotype isolates. The simplest interpretation of these findings is that ocular tropism is a derived state that has evolved only once and is defined by the tropism-conferring genomic variation specific to the branch leading to this lineage. Ocular tropism in this context refers to association of particular *C. trachomatis* strains with both: isolation from the eye and clinical signs of trachoma. Although candidate functional markers of tropism have been reported (reviewed in refs 11, 12, 13), the problem inherent to such studies is differentiating functionally significant variation from natural segregation of polymorphisms that have arisen through genetic drift and are therefore association markers of the classical ocular lineage. In addition, there are a small number of reports of trachoma associated with strains with UGT *ompA* genotypes^14^,^15^,^16^,^17^.

Australia is the only developed country where trachoma remains endemic, as well as being one of the few disease foci outside Africa where trachoma has been studied in detail^3^. In Australia, trachoma is exclusively associated with the Aboriginal population of remote regions^18^.

Here we report the first genome sequences of *C. trachomatis* isolates taken from ocular swabs of Australian patients with clinical trachoma. The isolates originate from a study of ocular *C. trachomatis* in Aboriginal children and UGT *C. trachomatis* in their mothers (termed the Mother–Child study), conducted in the north of the Australian Northern Territory (NT) between 1985 and 1993 (ref. 19). The study sites were remote Aboriginal communities where the regional trachoma prevalence was 12–28% in 0- to 14-year olds^20^, which fulfills the World Health Organization (WHO) definition (>5%) of trachoma endemicity. The study yielded cultured and stored *C. trachomatis* isolates. Little genomic data exist for *C. trachomatis* isolates causing trachoma from outside of Africa. To more fully understand the natural history of trachoma in Australia, and determine if it differs from other parts of the world, we performed genome sequencing on a selection of isolates. On the basis of genome-wide orthologous single-nucleotide polymorphisms (SNPs), the Australian isolates are not descended from the most common ancestor of the classical ocular clade, but rather are close relatives of urogenital isolates. This impacts current models regarding *C. trachomatis* evolutionary history and determinants of anatomical tropism as well as having implication for our attempts to eradicate trachoma.

## Results

### Culturing of historical trachoma isolates

The 1985–1991 Mother–Child study encompassed serological methods to type the collected isolates^19^. Results were largely consistent with expected associations between serovar and anatomical site of isolation (Supplementary Table 1). The presence of serovar B isolates in a significant proportion of urogenital samples was notable but not unprecedented, as there have been sporadic reports of the isolation of serotype or genotype B strains in urogenital specimens^21^,^22^,^23^,^24^,^25^,^26^,^27^,^28^,^29^,^30^. With growing knowledge of the genome diversity of *C. trachomatis* and the understanding that exchange of the *ompA* gene by recombination^8^,^9^,^10^,^31^,^32^,^33^,^34^,^35^,^36^,^37^,^38^ can mask our appreciation of that diversity; in 2013 we successfully revived, cultured and genome sequenced 12 ocular isolates from the Mother–Child study. Five of the revived isolates were fully documented and derived from research participants aged between 6 months and 9 years who displayed the follicles and/or papillae consistent with trachoma at the time of sample collection (Table 1). A further seven cultured samples could not be directly linked to individual participants, but were isolates taken from ocular swabs during that study. With the exception of one *ompA* genotype Ba isolate, which had been previously miscalled as serovar B, all *ompA* sequences reconstructed from our data were consistent with the serovar data derived from the Mother–Child study (Table 1).

### Whole-genome phylogeny shows novel trachoma lineages

**Figure 1:**
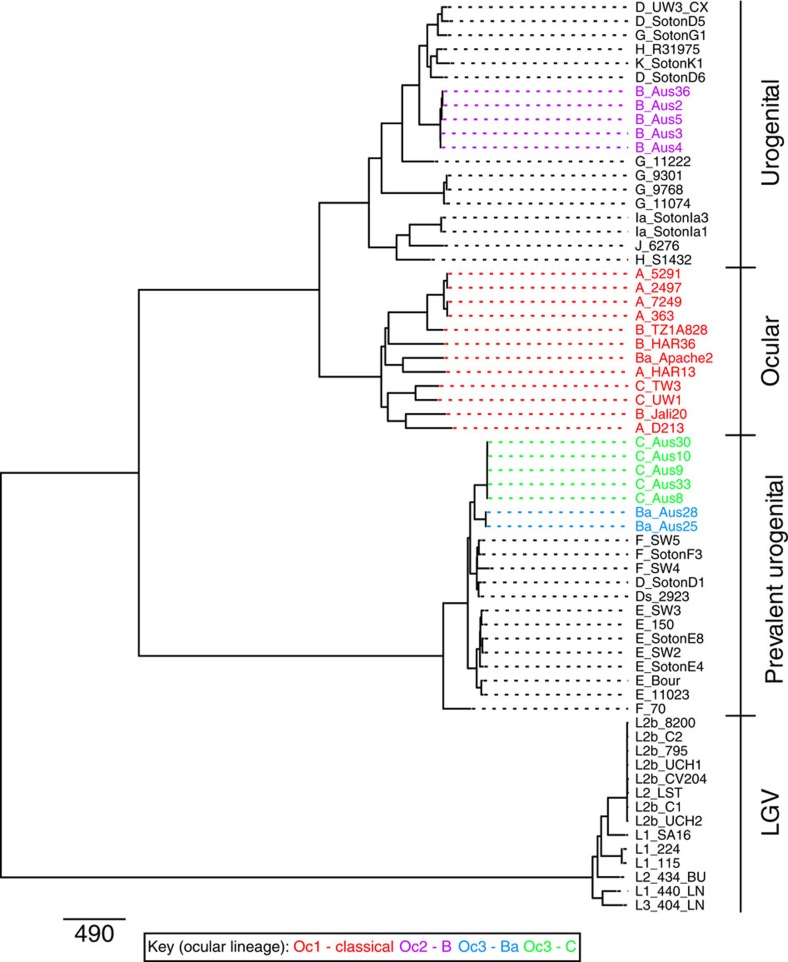
*C. trachomatis* chromosomal phylogeny. Maximum likelihood reconstruction of the phylogeny of *C. trachomatis* chromosomal sequences using orthologous SNPs, with inferred recombined regions removed. The *ompA* genotypes are included as the first letters in the designations of the isolates. The scale bar denotes number of SNPs. The established biovar-clusters are indicated on the right; with an ocular, a lymphogranuloma venerum (LGV) and two urogenital (UGT) clusters. The ocular clades are colour coded: Oc1 ‘classical ocular lineage' in red, Oc2 *ompA* genotype B Australian lineage in purple, Oc3 *ompA* genotype Ba Australian lineage in blue and Oc3 *ompA* genotype C Australian lineage in green. The novel ocular clades, Oc2 and Oc3 lie in clades that traditionally exclusively contain UGT strains.

The relationship of the sequenced isolates to a set of isolates, representative of the *C. trachomatis* global diversity, was determined using genome-wide orthologous SNPs^8^ (Fig. 1). To preserve the phylogenetic signal putative recombined regions were excluded from the analysis, as previously described^39^. However, with or without these regions, the trees shared essentially identical topologies (Supplementary Fig. 1a), indicating that the branching order is not affected by removal of recombined regions. The phylogenetic position of plasmids in the isolates was concordant with the chromosomal phylogeny (Supplementary Fig. 1b,c).

Surprisingly, the Australian isolates formed two separate lineages (which we have denoted Oc2 and Oc3), both falling outside the single classical lineage previously associated with trachoma (here termed Oc1; Fig. 1). Oc2 and Oc3 are unique, distinct and nested deeply within lineages that were previously occupied exclusively by urogenital *C. trachomatis* isolates (genotypes D–K; Fig. 1). Considering these Australian ocular isolates carry *ompA* genes that are characteristic of the classical ocular lineage and since it is well established that recombination occurs in *C. trachomatis*^8^,^9^,^10^,^32^,^34^,^36^,^40^, we used a sliding window-based approach to elucidate recombination events unique to the ancestry of our isolates. Using this approach, all the Australian ocular isolate genomes were compared against a set of published reference *C. trachomatis* genomes encompassing all three biovars. As expected from their position in the whole-genome species phylogeny, the chromosome of Oc2 clade isolates share extensive similarity to the D/UW-3 and G/11222 urogenital reference genomes, whereas the Oc3 clade isolates are most similar to the E/11023, F/SW4 and D/SotonD1 urogenital reference genomes.

### OmpA recombination boundaries

**Figure 2:**
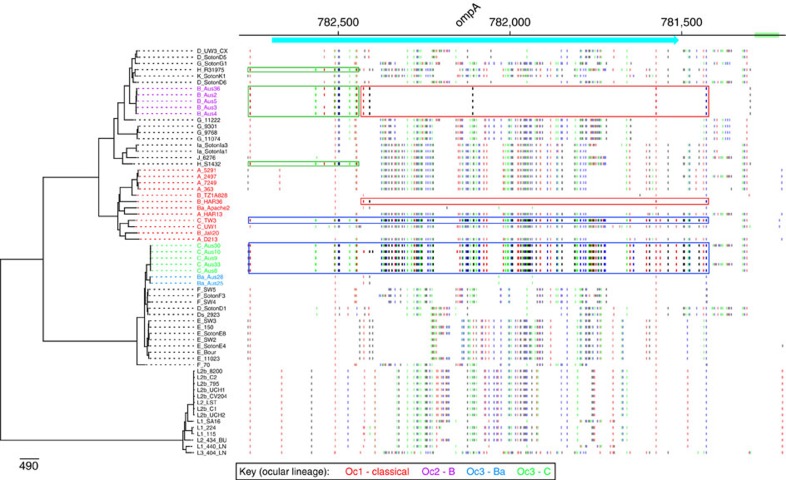
SNP patterns of the *ompA* gene in Oc2 and Oc3 genotype C isolates. SNP patterns in the region surrounding the *ompA* gene showing evidence for sharing of alleles between the Oc1 clade and the Oc2 or Oc3 lineages. The B/Jali20 genome (Genbank accession number NC012686) was used as the comparator genome. Genes are shown as blue boxes along the top with direction of transcription indicated by arrow heads, based on the B/Jali20 genome annotation. To the left, the chromosomal phylogeny (as in Fig. 1) indicates which genome is being compared with the B/Jali20 genome. Each horizontal line indicates a position where there is a SNP that separates the relevant genome sequence from the B/Jali20 genome sequence. The Oc2 clade has an ompA gene sequence most similar to the B/Har36 (indicated by red boxes), with the exception of the start of the gene, which is most similar to the two genotype H strains (indicated by the green boxes). The Oc3 *ompA* genotype C isolates have an *ompA* sequence most similar to that in the C/TW3 (blue boxes).

**Figure 3:**
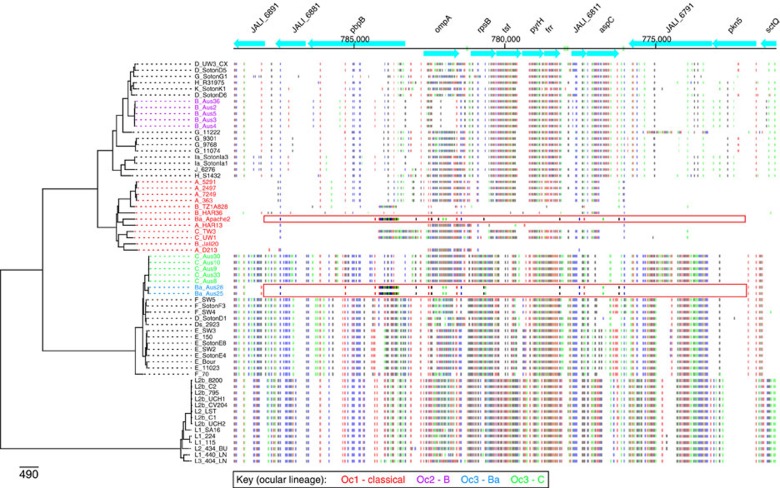
SNP patterns of the *ompA* gene in Oc3 genotype Ba isolates. SNP patterns in the region surrounding the *ompA* gene showing evidence for sharing of alleles between the Oc1 clade and the Oc2 or Oc3 lineages. The B/Jali20 genome (Genbank accession number NC012686) was used as the comparator genome. Genes are shown as blue boxes along the top with direction of transcription indicated by arrow heads, based on the B/Jali20 genome annotation. To the left, the chromosomal phylogeny (as in Fig. 1) indicates which genome is being compared with the B/Jali20 genome. Each horizontal line indicates a position where there is a SNP that separates the relevant genome sequence from the B/Jali20 genome sequence. The genotype Ba isolates in the Oc3 clade have a fragment including an *ompA* sequence that is most similar to the Ba/Apache2 strain (indicated by the red box).

Looking more closely at the *ompA* genes from these isolates it was evident that *ompA* from the five Australian genotype B strains (within Oc2) is itself a chimeric sequence, in which the 5′ end of the gene is identical to that of the *ompA* from H/R31975 and highly similar to *ompA* genes from other genotypes occupying closely branching positions on the tree (Fig. 2). The remainder of the Australian genotype B *ompA* gene is composed of a sequence that only differs from the *ompA* gene of B/HAR36 at one base position (Fig. 2 and Table 2). The boundaries for the recombined fragment including *ompA* in the Oc3 genotype C isolates correspond closely to the ends of the protein-coding sequence, whereas the fragment in the genotype Ba isolates extends on either side of *ompA* over a 11,405–16,142 bp region encompassing genes orthologous to ORFs *pkn5* to JALI_6881 in the B/Jali20 genome (Genbank accession number NC012686; Figs 2, 3).

### *PmpEFGH* genes are related to ocular orthologues

**Figure 4:**
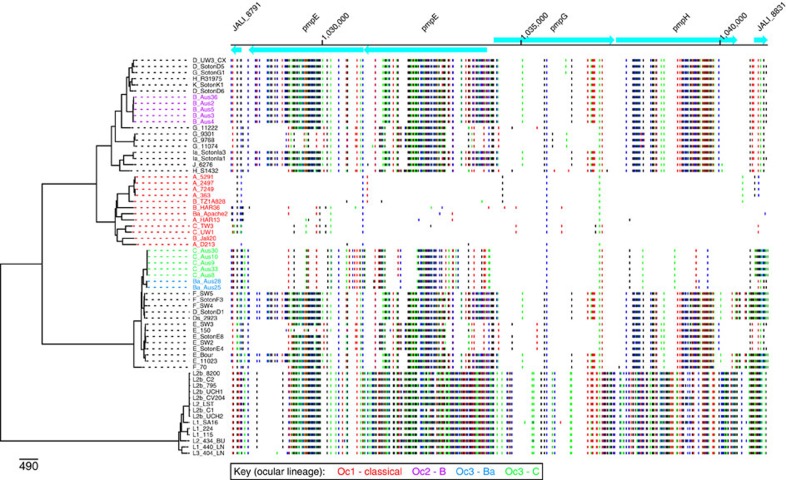
SNP patterns of *pmpEFGH* loci. SNP patterns in the region including the *pmpE*, *pmpF*, *pmpG*, *pmpH* genes showing evidence for sharing of alleles between the Oc1 clade and the Oc2 or Oc3 lineages. The B/Jali20 genome (Genbank accession number NC012686) was used as the comparator genome. Genes are shown as blue boxes along the top with direction of transcription indicated by arrow heads, based on the B/Jali20 genome annotation. To the left, the chromosomal phylogeny (as in Fig. 1) indicates which genome is being compared with the B/Jali20 genome. Each horizontal line indicates a position where there is a SNP that separates the relevant genome sequence from the B/Jali20 genome sequence. The sequence of the isolates in the Oc3 clade show the highest similarity to the Oc1 clade, with the most striking resemblance observed for the *pmpH* gene.

**Figure 5:**
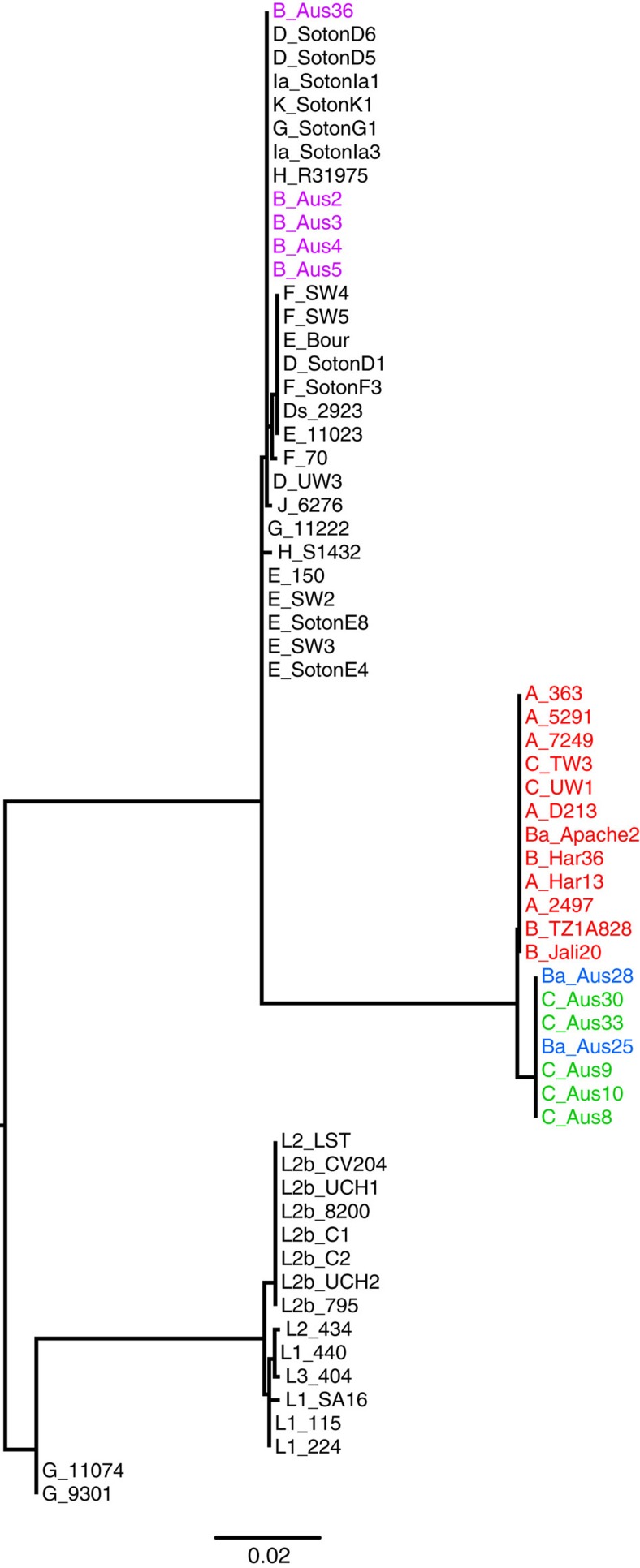
PmpH amino acid phylogeny. Maximum likelihood-inferred phylogeny of amino acid sequences for PmpH. The ocular clades are colour coded: Oc1 ‘classical ocular lineage' in red, Oc2 *ompA* genotype B Australian lineage in purple, Oc3 *ompA* genotype Ba Australian lineage in blue and *ompA* genotype C Australian lineage in green. The phylogeny shows a high similarity of the PmpH in Oc3 genotype Ba and C strains, with the PmpH in Oc1.

In addition to *ompA*, the only locus in the Australian trachoma isolates we could identify as being specifically similar to orthologues in the classical trachoma lineage was *pmpEFGH*. This cluster encodes polymorphic membrane proteins; associated with virulence^41^,^42^. The Oc3 *pmpE*, *pmpF*, *pmpG* and *pmpH* sequences were all more closely related to orthologues in the classical ocular lineage than in any known urogenital isolates (Figs 4, 5 and Supplementary Fig. 2a–c). However, it is notable that the Oc3 Pmp sequences are significantly diverged from any previously reported sequences, with 13, 11 and 3 unique amino-acid changes in PmpE, PmpF and PmpH, respectively. The amino-acid alignments are shown in Supplementary Fig. 3a–d.

Attempts to infer recombination events that have given rise to the Oc3 *pmpEFGH* locus yielded a complex picture, with evidence of mosaic structure both within the coding sequences and in flanking regions. For example, a 156 amino-acid stretch of PmpF is identical to PmpF from UGT isolates (Supplementary Fig. 3b). Also, the flanking regions for the genotype Ba and genotype C isolates in Oc3 differ, indicating that different recombination events have occurred in this region that nevertheless have yielded identical *pmpEFGH* loci (Supplementary Fig. 4).

### Genome-wide association study (GWAS) of the ocular phenotype

**Figure 6:**
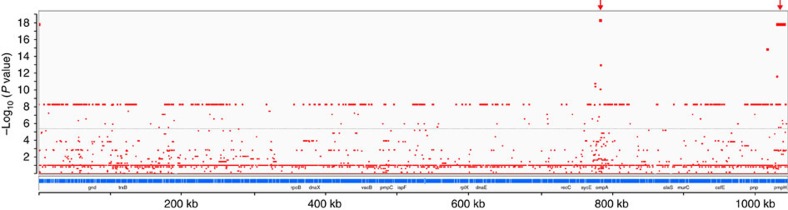
Genome-wide association study of the ocular phenotype. Manhattan plot showing genome-wide association data comparing ocular strains (Oc1, Oc2 and Oc3) against all other strains (UGT and LGV). Each red dot in the graph is a SNP. The *x* axis shows the genomic position and summary gene annotations according to the reference strain B/Jali20 (NC012686). The *y* axis shows the −log_10_ of the Bonferroni corrected *P*-value for association for each SNP. Red arrows show the two regions of very strong association with the ocular phenotype, containing *ompA* and *pmpEFGH*, respectively.

We used the orthologous SNP data in a GWAS, to assess whether there were other regions of the genome associated with trachoma that had not been identified by the sliding window approach. As expected, the *ompA* and *pmpEFGH* regions showed the strongest associations (Fig. 6). The large number of SNPs with a Bonferroni corrected −log_10_(*P*) 8.26 represent alleles that are associated with trachoma, but which are found only in the classical trachoma lineage and not in the Australian trachoma isolates. We reasoned that these are all linked due to common descent in a largely clonal lineage, and are unlikely to be functionally significant. It was concluded that the GWAS analysis provided no evidence for the functional involvement in trachoma of any loci apart from *ompA* and *pmpEFGH*.

### Other putative tropism loci

Allelic variation in the *tarP* gene, which encodes a type III secreted effector protein, has been proposed to correlate with *C. trachomatis* anatomical tropism when comparing African trachoma isolates with other pathovars of *C. trachomatis*^43^,^44^. However, this is not true of the *tarP* genes in our ocular isolates, which are consistent with those of the urogenital lineages they clustered within the genome-wide phylogeny. Notably, the only previous study^44^ of Australian trachoma-associated *C. trachomatis*, which included the sequence of a gene other than *ompA*, also found that *tarP* alleles in the Australian ocular genotype isolates did not cluster with *tarP* alleles from African trachoma isolates. Comparison of these *tarP* and *ompA* alleles with those found in our isolates showed identical *ompA* and *tarP* allele combinations.

Truncating mutations in the tryptophan synthesis genes (*trp* operon) have received considerable attention as characteristic markers of ocular tropism^23^. The *trp* operon in Oc2 is identical to that found in several common urogenital *C. trachomatis* isolates, for example, G/11222, F/SW4 and E/11023. Similarly, the *trp* operon in Oc3 is identical to that in the urogenital isolate F/70. There is no evidence for variations that would truncate the gene products.

### Urogenital and ocular serovar B form one lineage

It was of interest that genotype B strains were isolated in significant numbers from both the ocular and UGT sites in the Mother–Child study. In contrast, genotype Ba and C isolates were not obtained from the UGT site (see Methods for details). To investigate this further, six serovar B UGT isolates from the Mother–Child study were propagated and genome sequenced. The sequences were nearly identical to the Oc2 genotype B isolates, and there was no indication that the ocular and UGT isolates formed different group (Supplementary Fig. 5). This indicates that Oc2 was a lineage causing trachoma and being extensively transmitted sexually in the study area, at the time of sample collection.

## Discussion

This work presents the first genome sequencing of trachoma-associated *C. trachomatis* isolates from Australia. The isolates, which possess ocular-associated *ompA* variants, are from patients in trachoma-endemic regions presenting with a pathology and epidemiology typical of trachoma.

Given that previous species-wide multilocus sequence typing and genome sequence-based studies of *C. trachomatis* have consistently shown that isolates with ocular-associated *ompA* types fall within a single, monophyletic evolutionary lineage^8^,^9^,^10^, it was expected that the Australian trachoma isolates would also occupy a position within this classical ocular clade. However, phylogenetic reconstruction based on whole-genome SNPs show our isolates form two distinct lineages, each nested deeply within clades of global urogenital isolates and separated from the classical ocular lineage. The phylogenetic position of the plasmids in the Oc2 and Oc3 lineages is consistent with the whole-genome phylogeny, providing additional credibility regarding their evolutionary history. Previous studies have demonstrated concordance of chromosome and plasmid phylogenies^8^,^45^, although the Ba/Apache2 strains, which carries a plasmid identical to those found in urogenital strains (F/SW4, F/SW5/ D/SotonD1 and F/SotonF3) is an exception.

Our observations challenge the model suggested by previous studies that ocular tropism arose only once. The discovery that some Australian trachoma *C. trachomatis* genomes are almost entirely ‘urogenital' apart from the *ompA* gene suggests that the evolution of the ocular *ompA* alleles was functionally significant to the adaptation to the ocular niche and ability to cause trachoma. Remarkably, however, phylogenetic reconstruction has shown that the ‘ocular' *ompA* alleles themselves do not have a single origin^28^,^46^. Specifically, ocular *ompA* variants A and C define a group within the *ompA* sequence diversity that also includes the urogenital H, Ia, J and K alleles. In contrast, *ompA* alleles B and Ba are unrelated to A and C, but are related to E alleles (Supplementary Fig. 6). It is not possible to accommodate the triad of a single origin of ocular strains, a central role for *ompA* in conferring tropism, and the polyphyletic nature of ocular *ompA* alleles into an internally consistent model. The simplest interpretation and our new model for adaption to the trachoma biovar phenotype is that the ancestral virulence property of non-LGV *C. trachomatis* is essentially identical to current non-LGV UGT strains of *C. trachomatis*, in that they could cause urogenital infections and conjunctivitis. However, environmental conditions favouring long-term ocular infection lead to the parallel evolution of multiple *ompA* and *pmpEFGH* variants, which aid adaptation to that niche. These ‘ocular' alleles were added to the recombining gene pool and shared between strains through horizontal gene transfer. Thus, when environmental/social conditions favour the emergence of trachoma, the strains that have acquired these ‘ocular' alleles will be at a selective advantage and rapidly emerge as ‘trachoma' strains. Such a model has many parallels in bacterial evolution, particularly in antibiotic resistance. For example, the use of third-generation cephalosporins selects both the *de novo* appearance of point mutations that confer extended spectrum activity on β-lactamases, and also the radiation of lineages that have acquired the mutated genes from a recombining gene pool^47^. A corollary of this is that the apparent existence of the classical ocular lineage is a consequence of geographical sampling bias. We see no reason to assume that all trachoma-associated *ompA* alleles first arose in the classical ocular lineage.

It is important to note that there was strong evidence for functional tropism in *C. trachomatis* strains arising from the Mother–Child study. Although it is well known that UGT strains of *C. trachomatis* can cause conjunctivitis, primarily in adults and newborn infants, this does not have the characteristic clinical and epidemiological pattern of trachoma^48^,^49^,^50^. The ocular isolates analysed in this study are from a known trachoma endemic area, from young but not infant children exhibiting typical trachoma signs. Furthermore, we demonstrated a statistically significant association between serovar/strain and anatomical site of isolation (see Methods for details). We are therefore confident that our ocular isolates cannot be classed as urogenital strains causing conjunctivitis.

The identification of multiple lineages associated with ocular tropism will facilitate identification of functional markers of tropism that have previously been impossible to differentiate from drift. A search for loci in the Australian trachoma-associated *C. trachomatis* isolates with high similarity to those in the classical ocular lineage yielded only one hit apart from the *ompA*, the *pmpEFGH* loci in Oc3; and this was confirmed by the GWAS analysis.

The exact functions of the *pmp* genes remain to be determined, but certain protein features have been suggested to be of importance for pathogen–host interaction^33^,^35^,^51^,^52^,^53^, such as the clustering and conservation of tetrapeptide motifs, GGA(I,L,V) and FxxN in the N-termini. The Pmp variants in the Oc3 lineage display novel amino-acid changes and are evidently from a yet-unsampled horizontal gene transfer donor. However, only PmpE aa303 and PmpF aa385 affect GGA(I,L,V)/FxxN motifs (Supplementary Fig. 3a,b). These variants create new FxxN motifs that overlap with existing motifs, so the functional consequence is likely limited. Of more significance may be the presence of two motifs uniquely shared by the Oc1 and Oc3 lineages (PmpF FxxN aa384-7 and PmpH GAA(I,L,V) aa303-6, Supplementary Fig. 3b,d). This may be worthy of further study.

Supporting evidence for the involvement of the *pmpEFGH* genes in adaptation to the trachoma biovar phenotype can be seen in the genetic make-up of the unusual *C. trachomatis* isolate TW-448. TW-448 is a variant of the urogenital *ompA* genotype D termed genotype Da^54^. It was isolated from a trachoma case in an endemic trachoma region in Southern Taiwan, and is from a small collection of similarly anomalous isolates from a single Taiwanese family^15^. On the basis of a partial genome sequence, TW-448 has a predominantly ‘urogenital' genome, but the *pmpEFGH* locus has the appearance of being derived from the classical ocular lineage^34^,^35^ This locus is identical to the orthologue in isolate TW-3, which is an *ompA* genotype C trachoma isolate from Taiwan. This suggests recent horizontal gene transfer of *pmpEFGH* from the classical ocular lineage to a urogenital TW-448 precursor, and it was suggested by Gomes and co-workers that this locus may have a role in tropism^34^. Oc2, Oc3 and TW-448 therefore constitute three trachoma-associated lineages that, on the basis of genome-wide phylogeny are not located within the classical ocular lineage. The Oc2 lineage has acquired an ‘ocular' genotype B *ompA* gene, isolate TW-448 has acquired ocular *pmpEFGH* loci and the Oc3 lineage has acquired ocular-like *pmpEFGH* loci, and two different ocular *ompA* genes (genotype Ba and genotype C) in independent events. Therefore, sampling to date has revealed three independent *ompA* recombination events, and two independent *pmpEFGH* recombination events associated with evolving towards the trachoma biovar phenotype. Furthermore, a search of the literature and databases yielded no results describing horizontal transfer of other loci contributing to the adaptation to the trachoma biovar phenotype. Collectively, these observations constitute compelling evidence that particular sequence variants of the major surface proteins OmpA, and the Pmp family members are critical to the trachoma biovar phenotype. This is biologically plausible, as major surface proteins can mediate both interactions with the host, and also confer physical properties on the elementary bodies that could be connected with survival. It is intriguing that the Oc2 genotype B isolates are similar to the classical ocular lineage at *ompA* but not *pmpEFGH*. On the basis of anatomical distribution in the Mother–Child samples, and the virtually identical genomes of the ocular and UGT representatives, these have an intermediate tropism that allows both association with clinically typical trachoma and also extensive sexual transmission. We cannot rule out that other loci can contribute to tropism, but our study has given no indication as to the existence or nature of such loci.

A substantial body of evidence that truncating mutations in the *trp* operon are functional markers of ocular tropism has been accumulated^23^. Although our results do not provide additional evidence to support this model, a role for *trp* operon decay in adaptation to the ocular niche certainly cannot be ruled out. Decay of this operon may be a parallel evolutionary phenomenon that can occur in trachoma-associated lineages. Although this may contribute to adaptation, our evidence indicates it is not the sole determinant of tropism, and that adaption to the ocular niche can involve subsets of functional markers of adaptation.

The unexpected results of this study raise the question of whether the Oc2 and Oc3 lineages are representative of widespread trachoma strains in Australia. Several strands of evidence indicate that, in the case of Oc3 isolates, they are. First, the isolates in this study were from ocular swabs from young children with signs of trachoma, and thus represent what is typically seen in trachoma endemic areas. Second, there was a highly significant association between *ompA* genotype and anatomical site of isolation (see Methods for details), with trachoma cases in the Mother–Child study exclusively displaying classical trachoma *ompA* serotypes. Third, the *ompA* sequences from our *ompA* genotype Ba and C isolates are identical to the most prevalent *ompA* sequences previously described in Australian *ompA* genotype Ba and C trachoma isolates^49^,^55^. Finally, the findings of Lutter and co-workers concerning the *tarP* sequences^44^ are strongly indicative that our isolates are closely related to other reported Australian ocular isolates.

Previous studies of ocular isolates from Australia have not described *ompA* genotype B isolates, so the distribution of the *ompA* genotype B Oc2 lineage remains unknown. Chimeric *ompA* sequences identical to the variants in the Oc2 isolates from this study (Table 2) have been previously been described in *ompA* genotype B urogenital isolates from outside of Australia^23^,^24^,^28^. Our study yielded no classic trachoma lineage Oc1 genomes, reinforcing the model that the extensive signs of trachoma seen in this endemic region are attributable to a non-classical set of chlamydial ocular lineages.

In light of the theory that *C. trachomatis* may have been associated with primates long before the emergence of modern humans^56^, a possible explanation for the observed geographical distribution of the Australian strain genotypes may be that they represent strains that were carried with the first wave of migration out of Africa that populated Australia 70,000 years ago^57^, and have since then remained relatively isolated within the Australian Aboriginal population. However, given the close relationship of Oc2 and Oc3 to known UGT lineages, the accumulating evidence for frequent recombination by *C. trachomatis*, the known extensive interaction of Australian Aboriginal people with other human populations in historical times^3^,^58^, and the high prevalence of ‘conventional' UGT *C. trachomatis* strains in the contemporary Australian Aboriginal population^59^,^60^; there is no compelling evidence to support the notion that Oc2 and Oc3 represent early diverged lineages that were carried with the initial migration of people to Australia. However, more definitive information regarding the antiquity of Oc2 and Oc3 must await analysis of more isolates from around the world, and better understanding of the *C. trachomatis* molecular clock.

This study has public health implications. Trachoma remains a health burden in Australia, despite initiatives directed at elimination^18^,^61^. The WHO-defined target of elimination programmes is <5% prevalence^62^. Our results indicate that presence of residual ocular strains provide potential for gene variants assisting adaptation towards the trachoma biovar phenotype to be transferred to prevalent and successful UGT strains, and so facilitate trachoma persistence and re-emergence. The evidence that this has happened multiple times in Australia suggests that this is not a unique event. In addition, the apparent ability of *ompA* genotype B lineages to behave as either UGT or trachoma strains could also underpin re-emergence. This provides additional justification for reducing the burden of all *C. trachomatis* infections, ocular and UGT, in disadvantaged communities where trachoma re-emergence is possible, as well as continuing to address the social conditions that maintain trachoma endemicity.

## Methods

### Mother–Child study sampling

The sequenced isolates were derived from research conducted in the north of the NT of Australia between 1985 and 1993. The research project and interim results were described by authors L.V.A. and S.I.H. in a published long-form conference abstract^19^. In brief, the research entailed ocular sampling of children from Aboriginal communities in the dry ‘cattle country' of the NT, south-west of the town of Katherine; ocular sampling of Aboriginal children from three Aboriginal communities on the coast of the NT; cervical sampling of the mothers of the children from the coastal communities; and cervical sampling of women from a Darwin gynecology clinic. In addition, nasopharyngeal and rectal sites were sampled in some children. Essentially, all the sampling was performed by author F.P.D., who also prepared clinical notes associated with each sample. This included trachoma grading in accordance with the modified WHO system of 1981 (FPC system)^63^.

For ocular sampling, the swabs used were sterile, cotton tipped and aluminium shafted (Disposable Products Pty Ltd). Ocular swabs were taken from the upper subtarsal conjunctiva, unless upper lid eversion was not possible, in which case the lower subtarsal conjunctiva was swabbed after removal of any exudate. Each swab was put into a glass or polypropylene vial containing 1 ml sucrose phosphate transport medium (buffered sucrose phosphate (0.4 M) with 10% fetal calf serum, 100 μg ml^−1^ streptomycin sulphate, 20 μg ml^−1^ vancomycin hydrochloride and 2.5 μg ml^−1^ amphotericin B). The swab shaft was trimmed with sterile scissors, then the vial capped and stored at 4 °C for transport to the laboratory, and storage at −70 to –80 °C.

### Original cell culture and serotyping of *C. trachomatis* isolates

Isolates were initially cultured in 5 ml vials on McCoy cells^64^, by the method of Smeltzer *et al*.^65^ After storage at −70 °C for between 2 weeks and 3 years, cultures were serially passaged on McCoy cell monolayers in 5 ml vials, with infectivity of the monolayer being assessed after 72 h incubation using an inverted microscope. Passage, in increasing volumes, was continued until infectivity either reached 40–80% or failed to increase after at least five passages. The monolayer from the final flask was divided into two portions: one was resuspended in 1 ml growth medium and stored at −70 °C, and the other resuspended in 0.05 ml PBS with 0.02% (v/v)) formalin and stored at 4 °C until used for serotyping.

All serotyping procedures were performed in late 1980s and early 1990s. The isolates were serotyped by the micro-IF procedure of Wang *et al*.^5^, using the ‘*Chlamydia trachomatis* monoclonal antibody serotyping kit for all 18 serovars' (Washington Research Foundation), largely in accordance with the manufacturer's instructions. Fluorescence from the final reaction with sheep anti-mouse polyvalent immunoglobulin conjugated with fluorescein isothiocyanate (Silenus, ICI Diagnostic Division), diluted 1:30 in PBS, was read using an epifluorescence microscope. Serotypes were determined by comparing the observed fluorescence patterns with prototype strain reactions, as specified by the kit manufacturer.

The outcomes of the sample collection, culturing and serotype determination are shown in Supplementary Table 1. It can be seen that there was a significant correlation between anatomical site of isolation and serovar (Fisher's Exact Test and *χ*^2^ *P*-value <10^−7^). These were calculated using a 2 × 2 contingency table from the data in Supplementary Table 1, in which isolates were classed as being ‘ocular' or ‘UGT' serotypes, and sites of isolation either ‘ocular' or ‘UGT'. Regarding the potentially ambiguous data, ‘D/D′' was classed as a urogenital serotype, the rectum was classed as a UGT site of isolation, and the nasopharynx was classed as an ocular site of isolation. Serotype C and Ba isolates were found exclusively in non-UGT samples, whereas serovar B isolates displayed less anatomical specificity, with 7 isolates being derived from cervical swabs as well as 43 from ocular swabs in trachoma endemic areas. Conversely, the UGT serotypes were found predominantly in UGT samples.

In 2012, we commenced an initiative to sequence the genomes of trachoma-associated *C. trachomatis* from this study. All available hard copy material associated with study activities in the 1980s to 1990s was extracted from the Menzies School of Health Research archives and inspected. This yielded the original hand-written clinical notes prepared by author F.P.D., as well as the culturing and serotyping results (Supplementary Table 1).

We endeavoured to culture frozen isolates from ocular swabs. Isolates in the form of frozen ‘antigen reserve' cultures were physically located and their identities confirmed. This was performed primarily by authors P.A. and S.I.H. S.I.H. performed the culturing and serotyping in the 1980s and 1990s, and curated the culture collection, so there was considerable continuity of knowledge and expertise. To minimize the probability of sequencing isolates associated with perinatal conjunctivitis or adult follicular conjunctivitis, we confined our search to isolates from research participants between the ages of 6 months and 10 years. Seventeen frozen cultures of isolates that met the above criteria were transported to the laboratory of author I.N.C. Author P.A. attempted to culture all of these, to obtain sufficient genomic DNA for sequencing.

### Recent cell culturing

Frozen isolates were revived and propagated in McCoy cell (American Type Culture Collection) monolayers. The cells were grown in DMEM supplemented with 10% fetal calf serum. The inocula were centrifuged onto the confluent monolayer at 750*g* for 30 min at room temperature. Infected cultures were grown in DMEM supplemented with 10% fetal calf serum plus cyclohexamide at 1 μg ml^−1^ and gentamicin at 20 μg ml^−1^, for 72 h. *C. trachomatis* inclusions were identified using a phase contrast microscope. Isolates were propagated until high level of infection (80–100% of cells) was achieved. Isolates were passaged through increasing volumes, starting in 96-well plates, via 24-well and 6-well plates, to 25 cm^2^ tissue culture (T25) flasks. At the plate stages, isolates were cultured on individual plates to minimize risk of mix-ups and contamination. In the flask stage, each isolate was handled separately.

Infected monolayers were detached from flasks using a cell scraper and centrifuged at 3,000*g* for 10 min. The infected cell pellet was resuspended in cold 10% PBS and homogenized using glass beads and vortexed at full speed for a minute, to open cells and release EBs. Cell debris was removed by centrifugation at 250*g* for 5 min, and the supernatant containing partially purified elementary bodies was mixed with an equal volume of phosphate buffer containing 0.4 M sucrose (4SP), before storage at −80 °C. Culturing was successful for five isolates: Aus25, Aus28, Aus30, Aus33 and Aus36. Aus25 was recorded as ‘West Coast, serotype Ba', Aus28 and Aus36 as ‘West Coast, serotype B', and Aus30 and Aus33 as ‘Tropical Coast, serotype C'. These were unambiguously identified and matched with original clinical notes.

In addition, another seven stored isolates were successfully cultured by author H.M.B.S.S. These were frozen in T25 culture flasks. These vessels had been originally labelled only with serotype designations, and not with study-related code-numbers. Therefore, although these isolates are certainly derived from the study (no other *C. trachomatis* are stored at the Menzies School of Health Research), they could not be matched to clinical or epidemiological data. These isolates have been named Aus2, Aus3, Aus4, Aus5 (serotype B), and Aus8, Aus9 and Aus10 (serotype C). The possibility that these isolates overlap with isolates Aus25, Aus28, Aus30, Aus33 and Aus36 cannot be ruled out. Although it is unlikely, this possibility was taken into account in the interpretation of the results.

Six serovar B isolates from cervical samples (Aus40, Aus41, Aus42, Aus43, Aus44 and Aus45) were revived and cultured as described above. These isolates were used to provide context for the ocular serovar B isolates.

### DNA extraction and sequencing

For each isolate, three T25 flasks at 80–100% infectivity were used for DNA extraction. DNA was extracted and purified using a Promega Wizard Genome Purification kit (Promega UK) according to the manufacturer's protocol. The quality of the DNA was assessed by agarose gel electrophoresis. Isolates were sequenced using the Illumina HiSeq or MiSeq platform. The protocol used either 100-base reads (Aus2, 3, 4, 5, 8, 9, 10) with 250–300 base inserts on the HiSeq platform, or 150-base reads (Aus25, 28, 30, 33, 36, 40–45) with 250–300 base inserts on the MiSeq platform. These samples were among 96 samples multiplexed in one lane. The sequencing runs provided an average of 6 million reads (658 Mb) per isolate. The sequence read archive (SRA) numbers and accession numbers for all strains used in this study are presented in Supplementary Table 2.

### Phylogenetic reconstruction

A whole-genome alignment from the work by Harris *et al*.^8^ was used to create a consensus sequence in Ugene. For all samples, the reads were mapped against the consensus sequence using SMALT, resulting in an average genome coverage of 99.125% and average depth of coverage of 338x. For samples where published sequences were used, synthetic reads were created *in silico* from the whole-genome sequences (100 bp reads, with 300 bp insert, for each base along the genome). SNPs were identified using a combination of samtools mpileup and bcftools. Filters were applied so that only high-quality SNPs were accepted, where sites were excluded if the SNP quality score was less than 30 or the SNP was present in less than 75% of the reads^66^. A new alignment was created from the mapping data and used for phylogenetic reconstruction using RAxML v7.0.4 under a Generalized Time Reversible model of evolution with a γ correction for among-site rate variation with four rate categories. A previously described iterative recombination removal method was applied to reduce the effect of recombination on the phylogeny^67^. In the phylogeny, the three separate ocular clades were designated: Oc1 (classical trachoma lineage), Oc2 (novel trachoma lineage composed of Australian serovar B strains) and Oc3 (novel trachoma lineage composed of Australian serovar Ba and C strains).

### Horizontal gene transfer from Oc1 trachoma lineage

Sequence identity to a known set of strains was interrogated using mapping. A set of 15 published full genomes representative of the *C. trachomatis* diversity were chosen and aligned with Mauve^68^ and manually error corrected, as previously described^8^. The query sequence was mapped against an un-gapped version of this alignment using SMALT (as above). A sliding window approach (defaults were: whole genome: block size of 5,000 and 1,000 bp sliding window or, for genes: block size of 10 and a 5 bp sliding window) used mapping coverage as a proxy for similarity. The mapping depths were overlaid using the alignment and a consensus ‘top match' identified the largest contiguous segments with mapping depth >90% of the maximum at each position. To identify regions that may have been derived from the classical ocular lineage, we searched for regions in which classical ocular reference genomes transitioned to being the most similar to the Oc2 or Oc3 isolates. Such regions were manually inspected to determine the number of SNPs involved in conferring this aberrant relative similarity. Only regions of similarity in which the signal for putative recombination was >5 SNPs per 1,000 bp window were considered for further analysis. The regions identified in this scan were further analysed in detail using the SNP barcode method described below.

### SNP barcoding

For all samples, reads were mapped against the B_Jali20 reference strain (NC012686) using the mapping protocol described above. The resulting SNP matrix was used together with the phylogenetic tree described above, to generate the figures of SNP barcodes with reference to B/Jali20.

### Genome-wide association study

Short read data from all strains in Fig. 1 were mapped against the B_Jali20 reference strain (NC012686) using SPANDx v2.6 (ref. 69), with default parameters. The resulting orthologous SNP matrix was used as input for a microbial GWAS using PLINK^70^, with the phenotype of interest being isolation from a trachoma case. Strains with an association with trachoma (Oc1, Oc2 and Oc3) were treated as the group displaying the phenotype, and compared against all other strains in the study. Bonferroni correction for multiple testing was applied, with an adjusted −log_10_(*P*)-value threshold for significance of 5.44. GWAS results were displayed using the Integrative Genome Viewer (IGV) software (Broad Institute).

### Ethics

Research from 1985 to 1993 was approved by the Institutional Ethics Committee of the Menzies School of Health Research, and participants provided informed consent. The Human Research Committee of the Northern Territory Government Department of Health provided a letter indicating that the genome sequencing did not require further ethical clearance.

## Additional information

**Accession codes:** The short-read data in fastq format for all strains described in this study have been deposited in the National Center for Biotechnology Information (NCBI) Sequence Read Archive (SRA) database, and accession codes are provided in Supplementary Table 2.

**How to cite this article:** Andersson, P. *et al*. *Chlamydia trachomatis* from Australian Aboriginal people with trachoma are polyphyletic composed of multiple distinctive lineages. *Nat. Commun.* 7:10688 doi: 10.1038/ncomms10688 (2016).

## Supplementary Material

Supplementary Information Supplementary Figures 1-6, Supplementary Table 1-2 and Supplementary References

## Figures and Tables

**Table 1:** Isolate characteristics and associated clinical data.

**Sample no**	Serovar*	***ompA*** **genotype**†	**Age (years)**‡	**Trachoma grading**§
Aus25	Ba	Ba	6 Months	F_1_ P_0_ C_0_
Aus28	B	Ba	9 Months	F_0_ P_2_ C_0_
Aus30	C	C	15 Months	F_1_ P_2_ C_0_
Aus33	C	C	15 Months	F_0_ P_2_ C_0_
Aus36	B	B	9 Years	F_1_ P_2_ C_0_
Aus2||	B	B	—	—
Aus3||	B	B	—	—
Aus4||	B	B	—	—
Aus5||	B	B	—	—
Aus8||	C	C	—	—
Aus9||	C	C	—	—
Aus10||	C	C	—	—
Aus40¶	B	B	18 Years	—
Aus41¶	B	B	20 Years	—
Aus42¶	B	B	32 Years	—
Aus43¶	B	B	24 Years	—
Aus44¶	B	B	25 Years	—
Aus45¶	B	B	24 Years	—

The serovar shown was determined by microimmunofluorescence test using monoclonal antibodies as part of the standard protocol in the original sampling study and provided as a reference for the serovars determined in this study. Trachoma grading was carried out according to the modified WHO system of 1981 (FPC system)^63^. For seven isolates, no clinical data were available.

Samples Aus40-Aus45 were isolated from cervical samples.

^*^Serotyping determined using monoclonal antibodies as part of the original sampling study.

^†^*ompA* genotype determined from whole-genome sequence data in the present study.

^‡^Age of patient at time of sampling.

^§^F=follicles, P=papillae, C=conjunctival scarring; all following a three graded scale.

^||^For these samples, no clinical data were available.

^¶^These samples were isolated from an urogenital site.

**Table 2:** Variations in the *ompA* gene.

***ompA*** **genotype (*n*)**	**Closest match**	**Nucleotide change**	**Amino-acid change**
B (5)	B/Har36 (this study)	C129T*	Synonymous
		A154G*	Thr 52 Ala
		A184G*	Met 62 Val
		G186T*	Met 62 Val
		T195C*	Synonymous
		T198A*	Synonymous
		A228T*	Synonymous
		C246T*	Synonymous
		A249G*	Synonymous
		G586A	Val 196 Ile
Ba (2)	Ba/Apache2 (AF063194)	A511G†	Ser 171 Gly
		C662T†	Pro 221 Leu
C (5)	C/TW3 (AF352789)	T569C†	Ile 190 Thr
		A571G†	Asn 191 Asp
		G972A†	Synonymous
		G1003T†	Ala 335 Ser
		A1063C†	Met 356 Leu

The trachoma-associated strains from Australia were compared with closest reference strains.

^*^These SNP changes in the *ompA genotype* B isolates correspond exactly with the sequence of a serovar H strain, and is likely the result of a recombination event creating a chimeric *ompA* sequence.

^†^These variations have been observed previously in trachoma patients in Australia.
