# Supplementary material for: Chlamydia trachomatis from Australian Aboriginal people with trachoma are polyphyletic composed of multiple distinctive lineages
Source: Nat Commun. 2016 Feb 25;7:10688. doi: 10.1038/ncomms10688 (PMC4773424; doi:10.1038/ncomms10688)
Supplement: Supplementary Information — Supplementary Figures 1-6, Supplementary Table 1-2 and Supplementary References [file ncomms10688-s1.pdf]

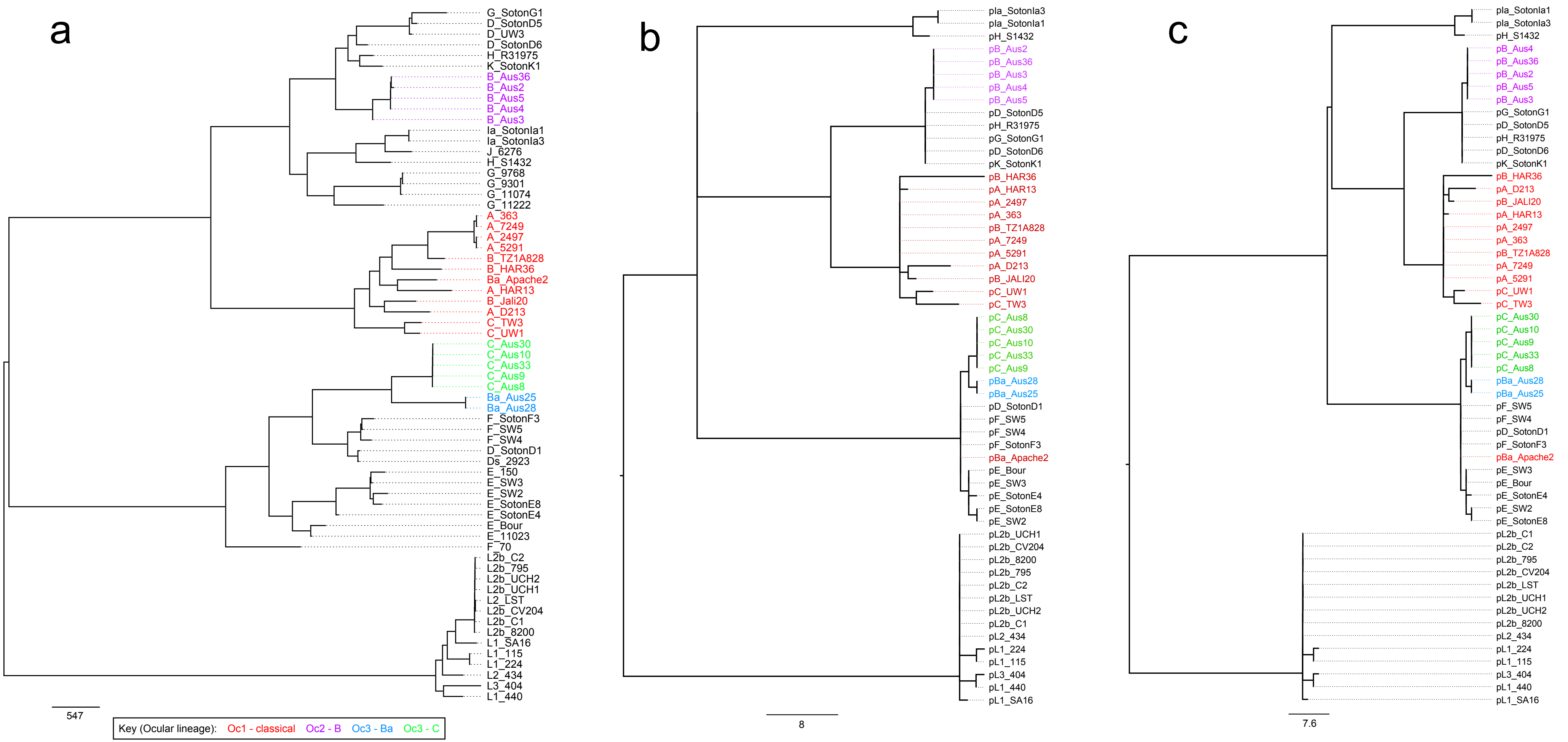

Supplementary Figure 1 | *C. trachomatis* chromosomal and plasmid phylogenies. Maximum likelihood reconstruction of the phylogeny of *C. trachomatis* sequences using orthologous SNPs. The *ompA* genotypes are included as the first letters in the designations of the isolates. The established biovar-clusters are indicated on the right; with an ocular, a LGV and two urogenital clusters. The ocular clades are colour coded: Oc1 “classical ocular lineage” in red, Oc2 *ompA* genotype B Australian lineage in purple, Oc3 *ompA* genotype Ba Australian lineage in blue and *ompA* genotype C Australian lineage in green. The scale bar denotes number of SNPs. (**a**) Chromosomal sequences with all orthologous SNPs. (**b**) Plasmid sequences with inferred recombined regions removed. (**c**) Plasmid sequences with all orthologous SNPs included. All trees are of the same general topology as Figure 1 in the main text, indicating that the chromosome and plasmid phylogeny are concordant for Oc2 and Oc3, and that removing putative recombined regions does not affect the conclusions.

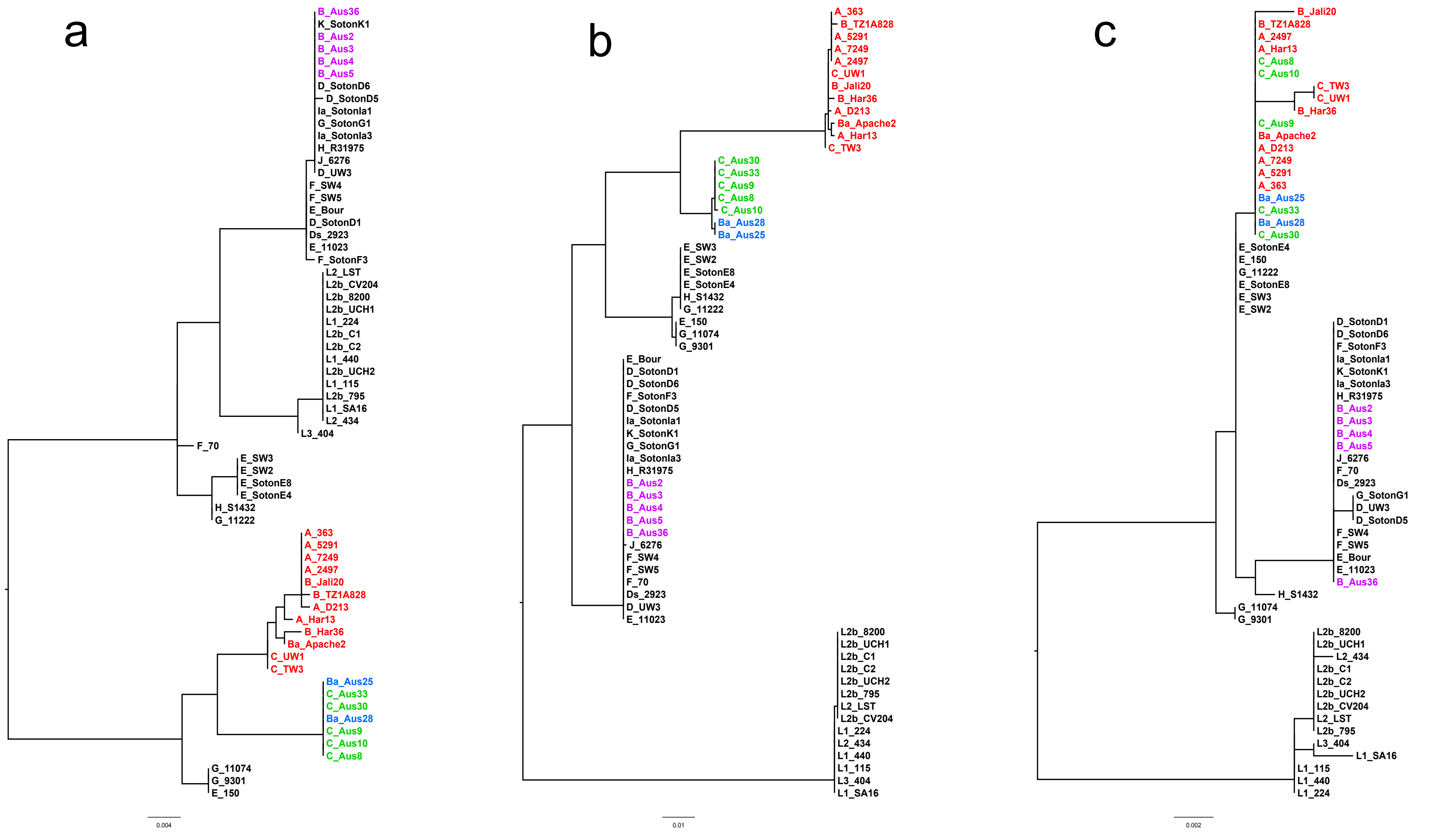

Supplementary Figure 2 | Maximum likelihood-inferred phylogeny of PmpE, PmpF and PmpG amino acid sequences.
(**a**) PmpE, (**b**) PmpF and (**c**) PmpG. The classical trachoma strains are labelled in red. The Australian ocular isolates sequenced in this work are labelled: Oc2 genotype B – purple, Oc3 genotype Ba – blue and Oc3 genotype C - green. For all three genes the Australian trachoma strains are more closely related to classical ocular strains than urogenital strains.

Supplementary Figure 3 | PmpE, PmpF, PmpG and PmpH amino acid alignments. The strains in the Oc3 lineage display unique amino acid changes in PmpE, PmpF and PmpH that have previously not been described. The GGA(I,L,V) and FxxN motifs are marked with red and blue boxes, respectively. (**a**) PmpE. (**b**) PmpF. (**c**) PmpG. (**d**) PmpH.

Supplementary Figure 3a | Amino acid alignment of *pmpE* (page 1/2)

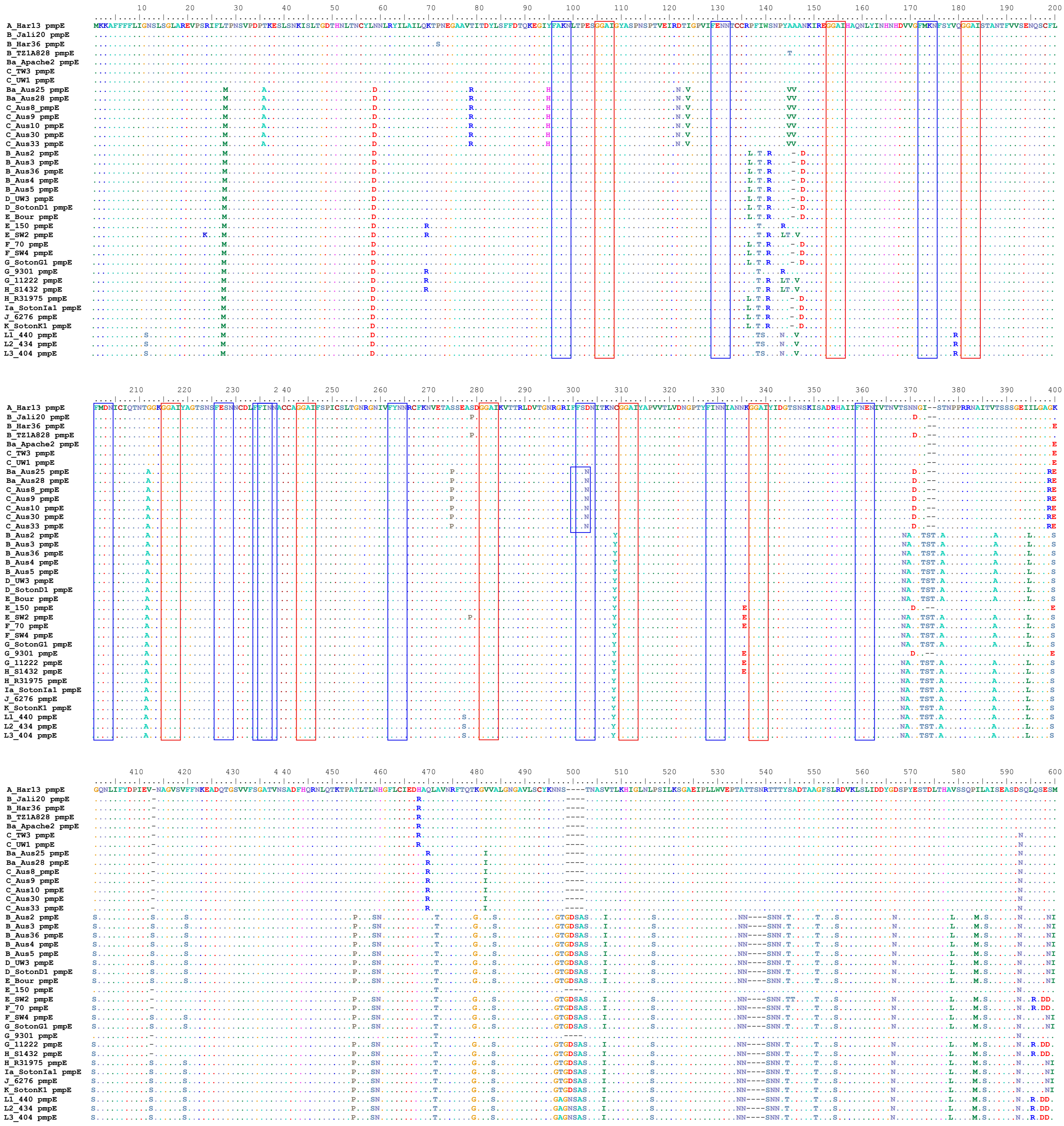

Supplementary Figure 3a | Amino acid alignment of *pmpE* (page 2/2)

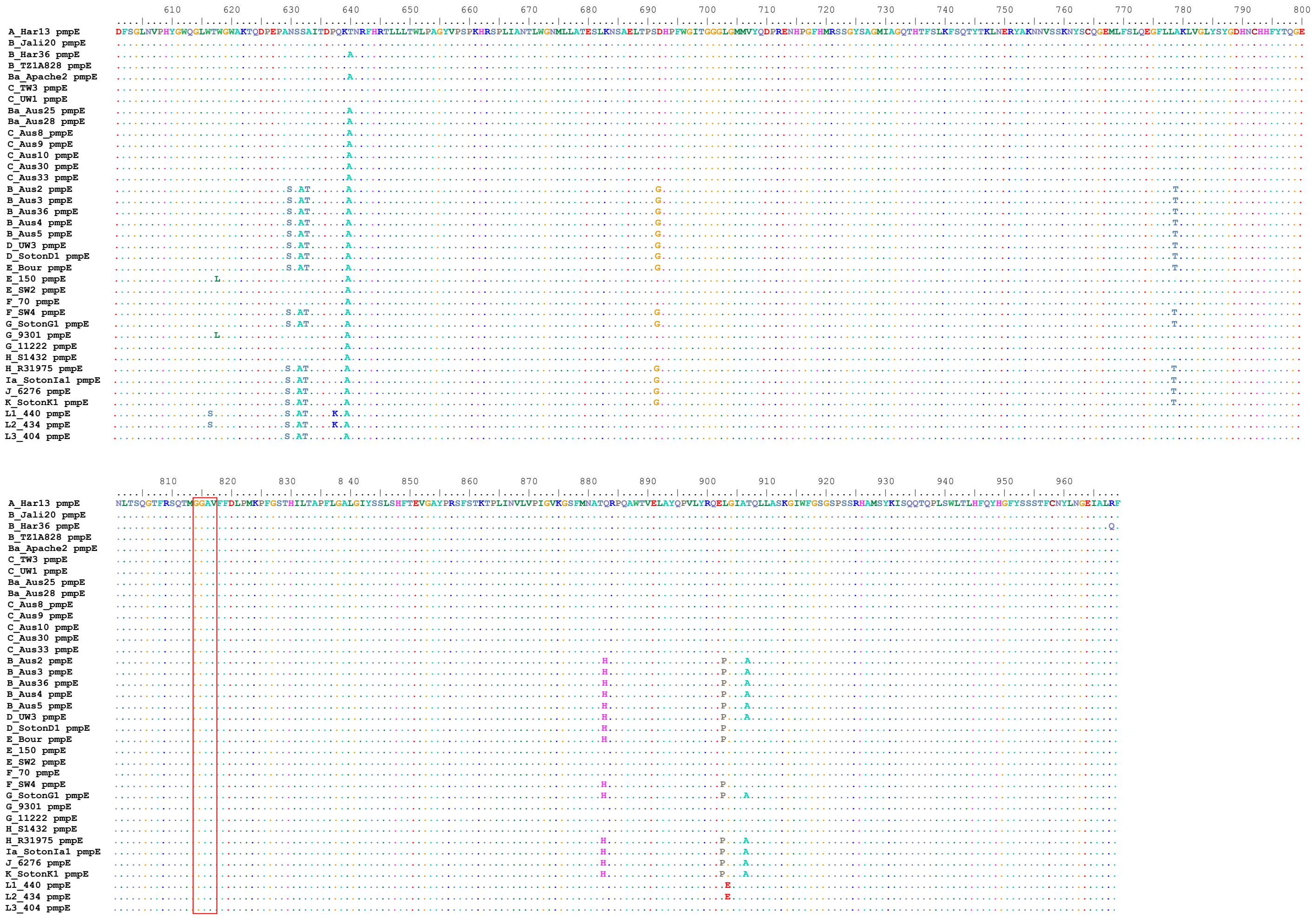

Supplementary Figure 3b | Amino acid alignment of *pmpF* (page 1/2)

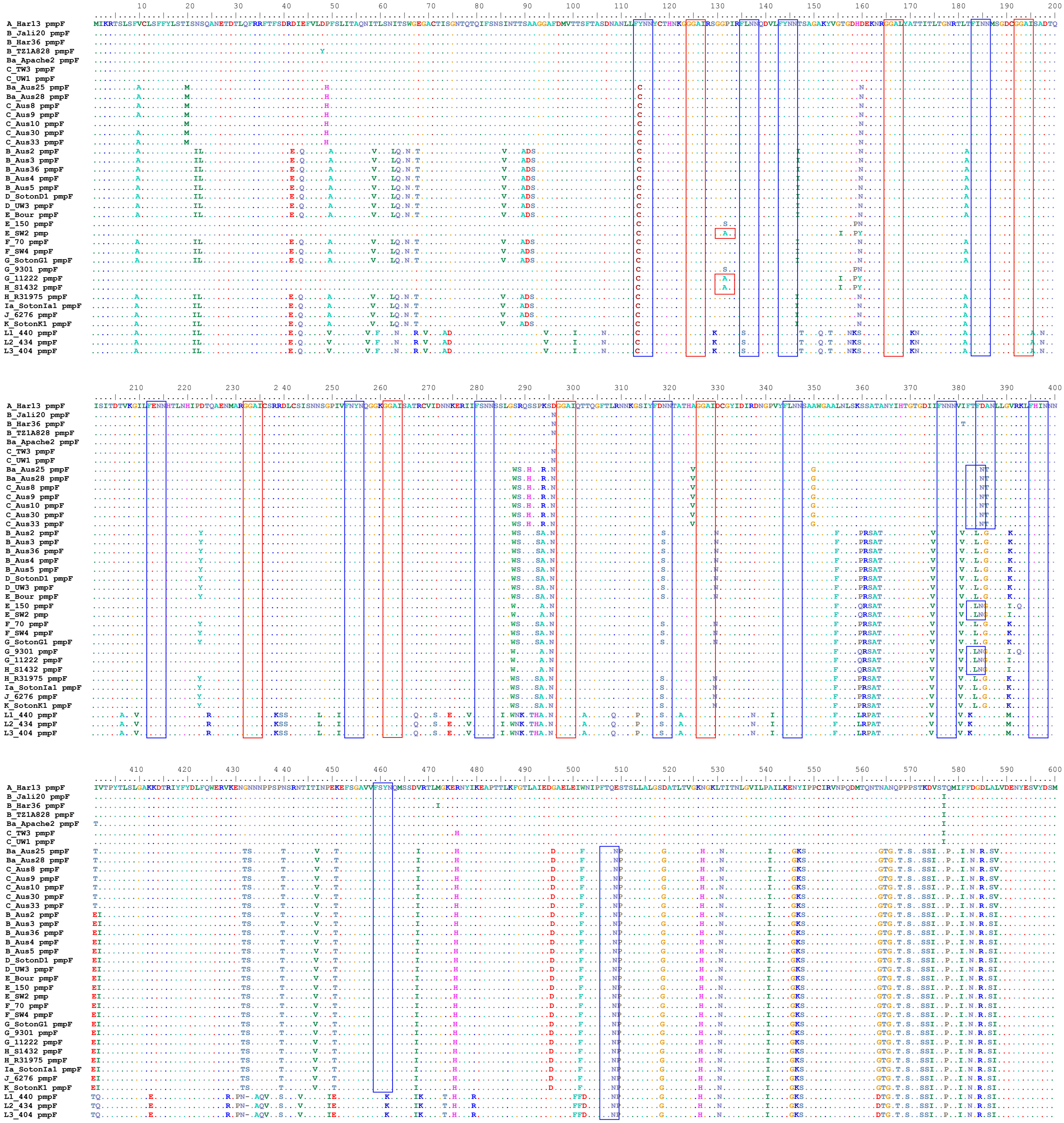

Supplementary Figure 3b | Amino acid alignment of *pmpF* (page 2/2)

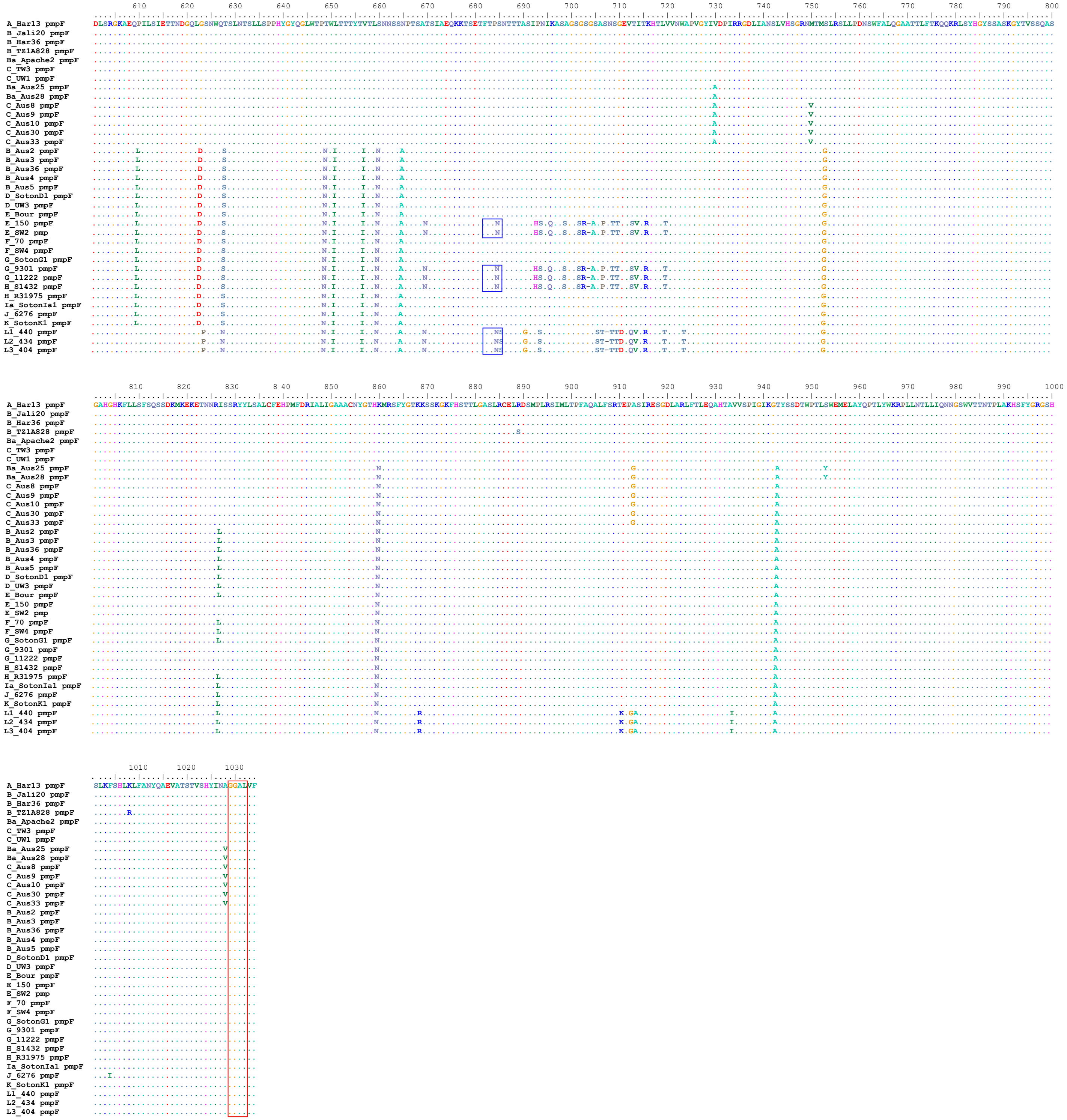

Supplementary Figure 3c | Amino acid alignment of *pmpG* (page 1/2)

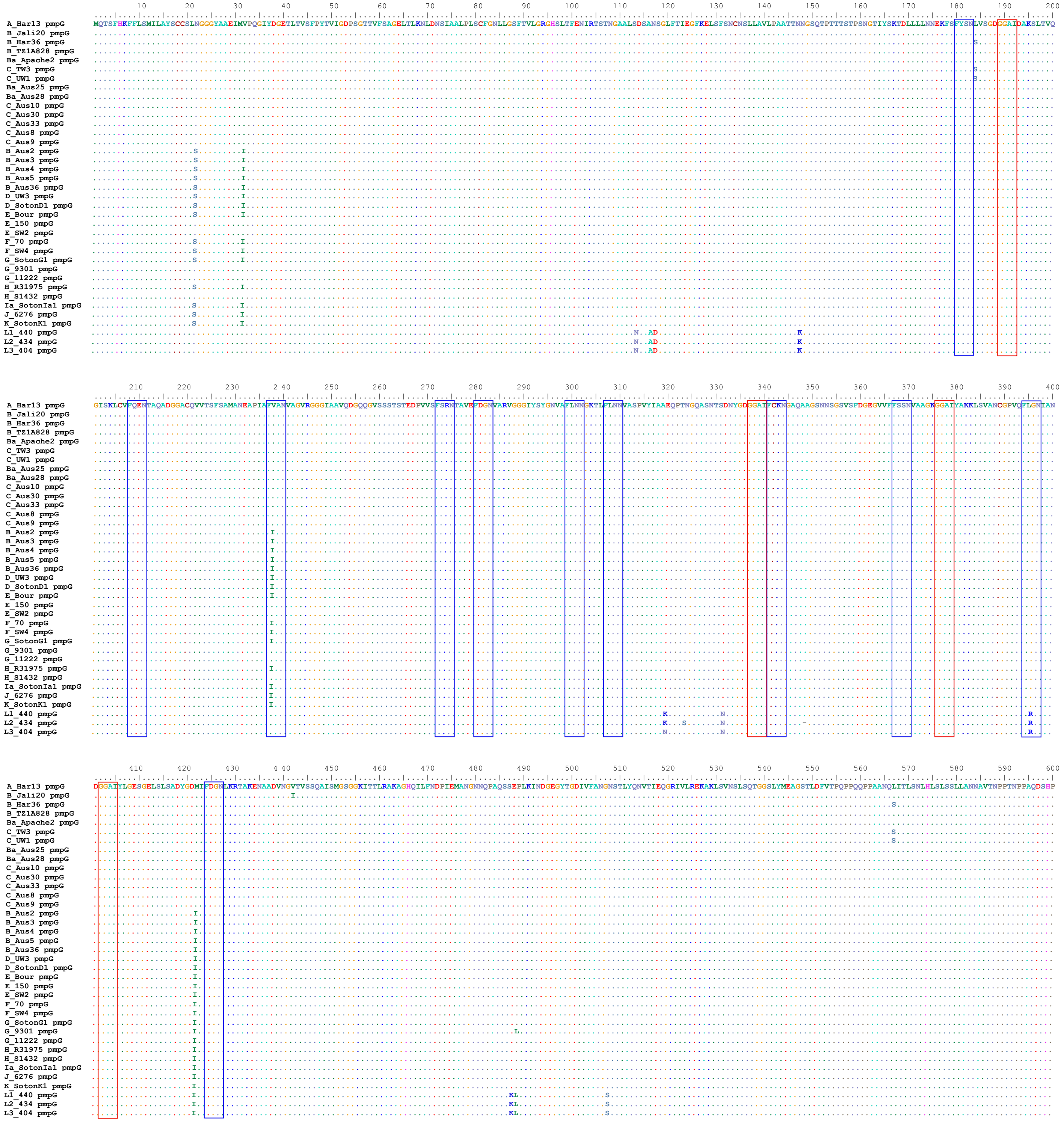

Supplementary Figure 3c | Amino acid alignment of *pmpG* (page 2/2)

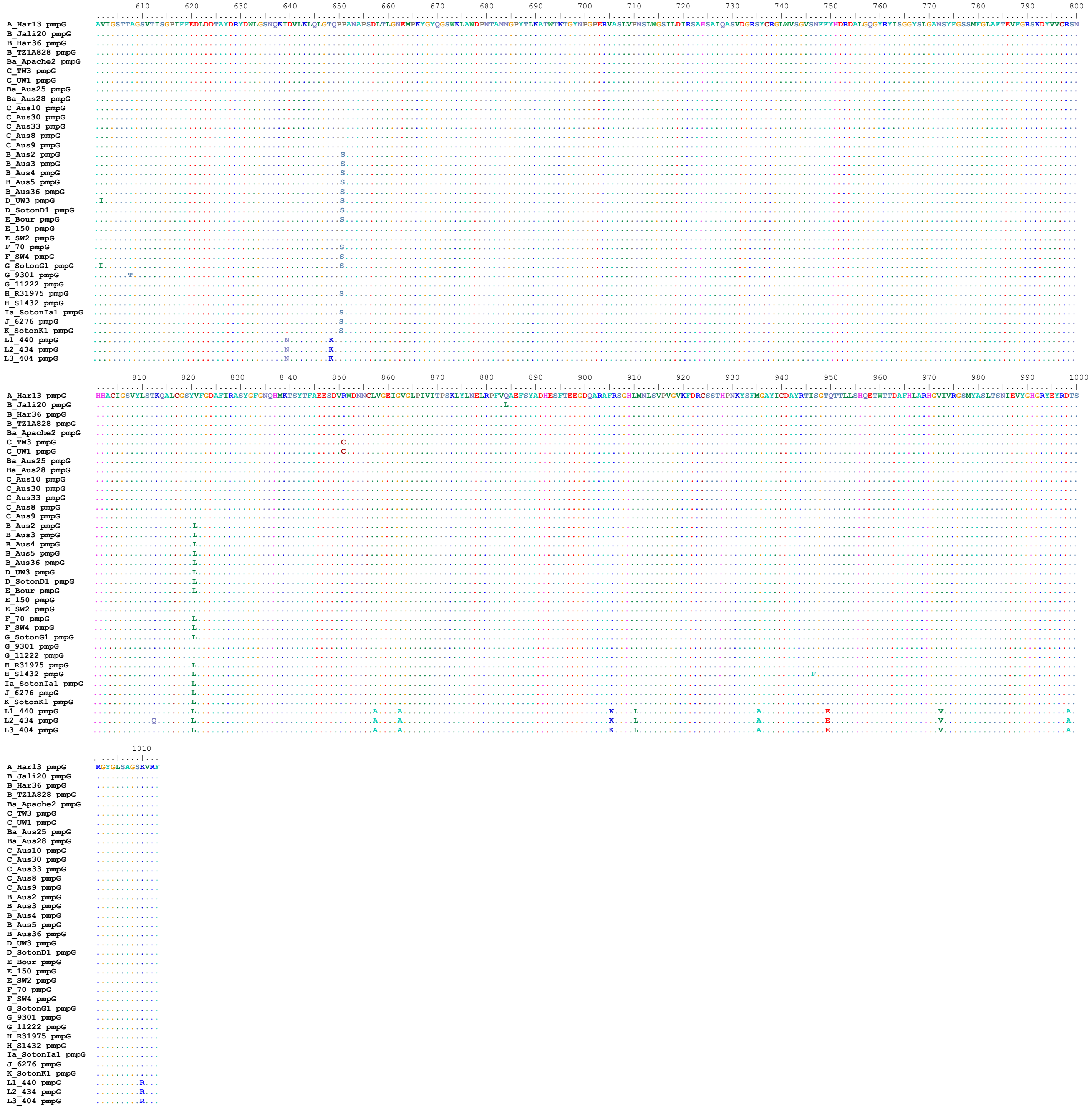

Supplementary Figure 3d | Amino acid alignment of *pmpH* (page 1/2)

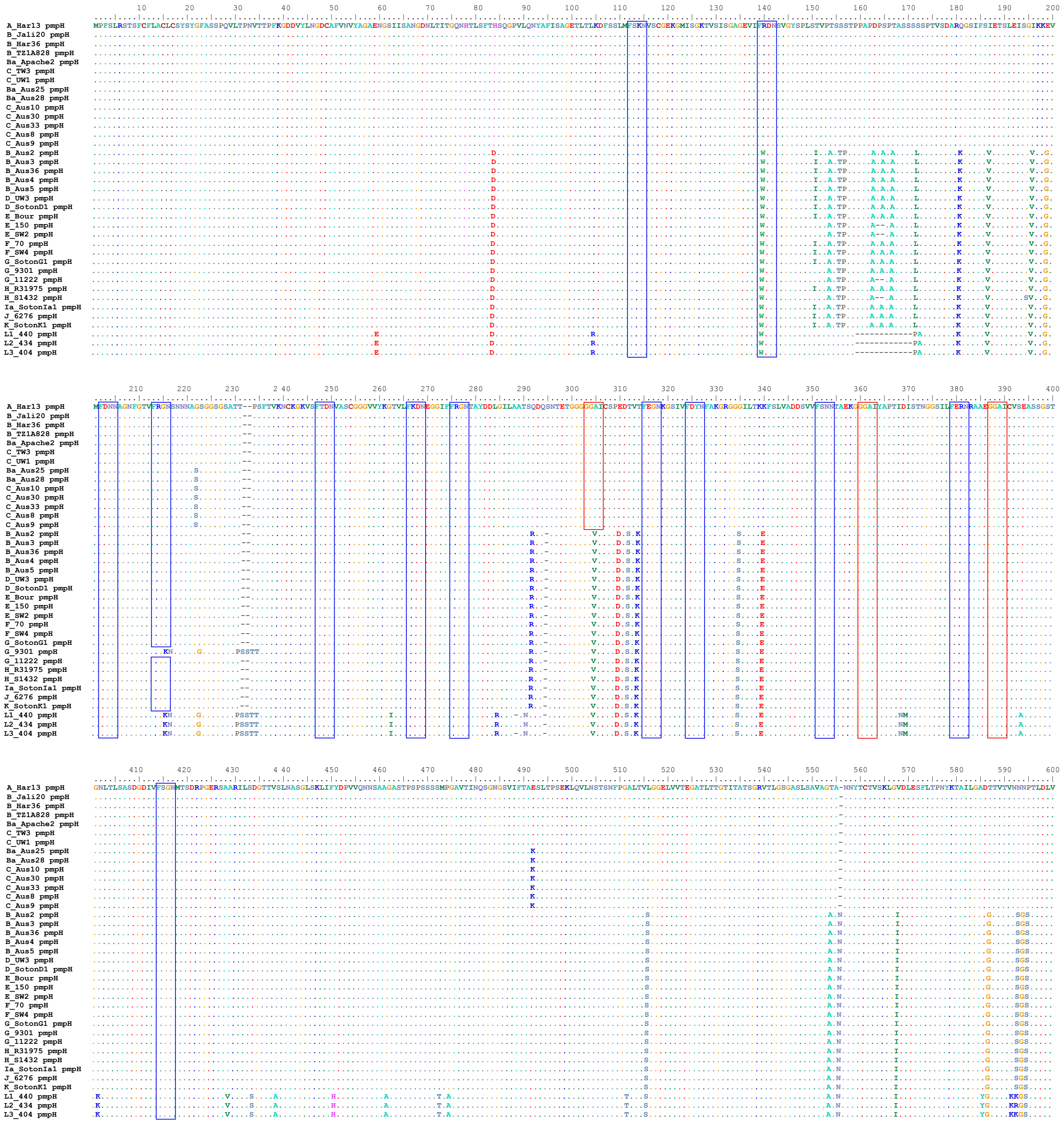

Supplementary Figure 3d | Amino acid alignment of *pmpH* (page 2/2)

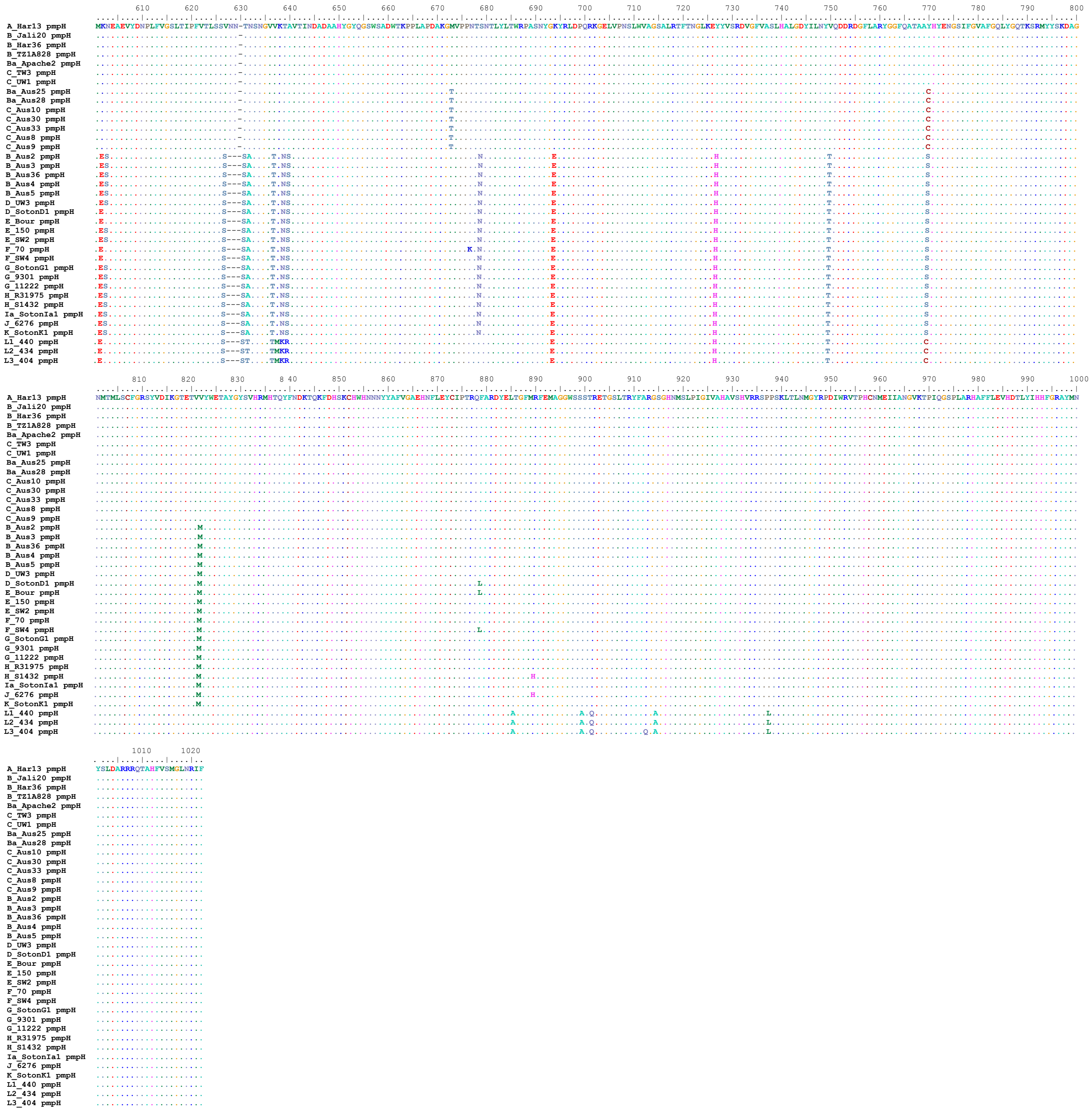

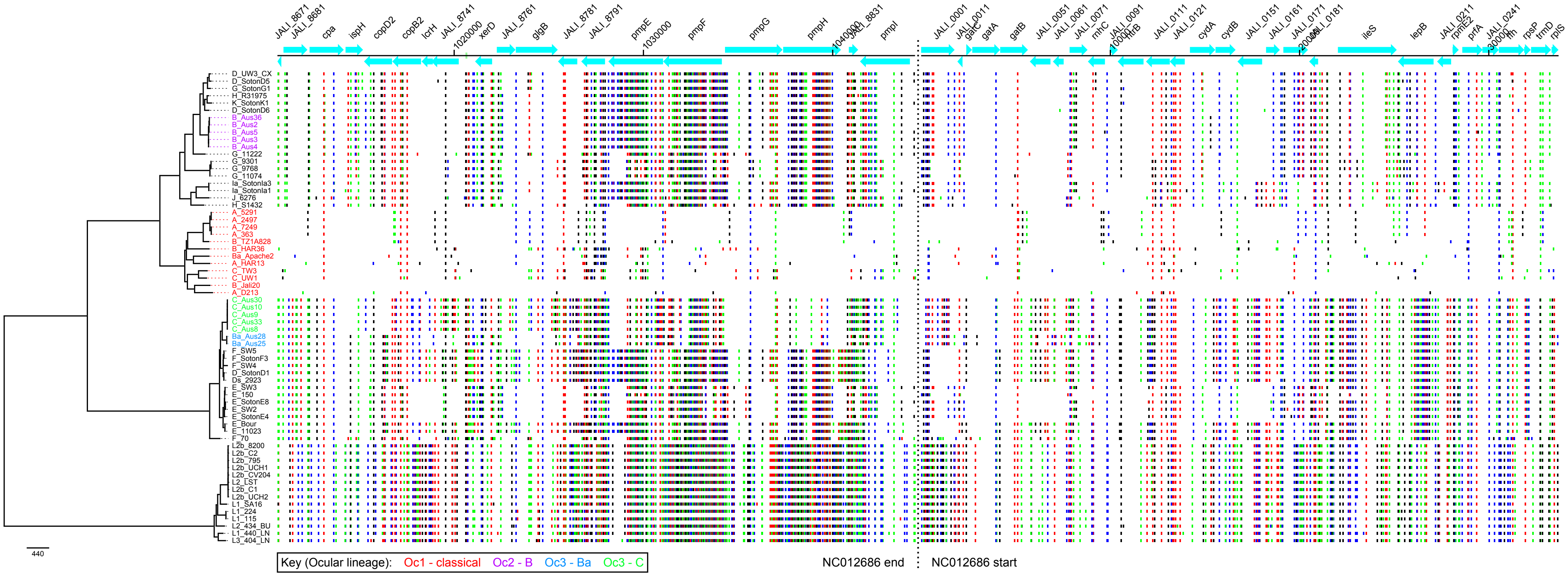

Supplementary Figure 4 | SNP patterns of extended *pmpEFGH* region. SNP patterns in an extended region compared with Fig 4, including the *pmpE*, *pmpF*, *pmpG*, *pmpH* genes and showing the mosaic nature of this genome segment. The B_Jali20 genome (Genbank accession number NC012686) was used as the comparator genome. Genes are shown as blue boxes along the top with direction of transcription indicated by arrow heads, based on the B_Jali20 genome annotation. To the left, the chromosomal phylogeny (as in Fig 1a) indicates which genome is being compared with the B/Jali20 genome. Each horizontal line indicates a position where there is a SNP that separates the relevant genome sequence from the B_Jali20 genome sequence. The dotted line marks the start/end break in the linearized genome of NC012686.

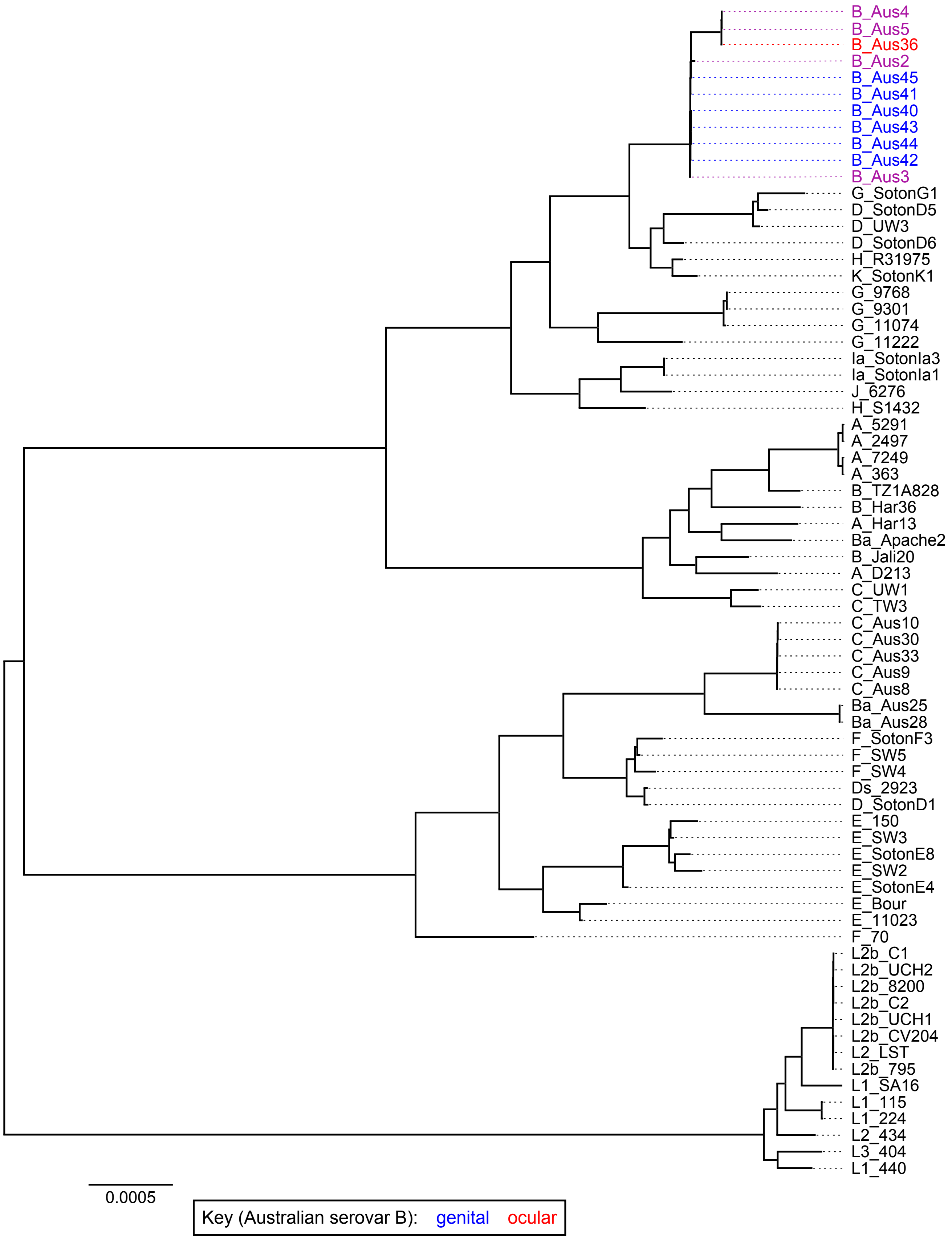

Supplementary Figure 5 | *C. trachomatis* chromosomal phylogeny showing Australian ocular and urogenital genotype B strains. Maximum likelihood reconstruction of the phylogeny of *C. trachomatis* sequences using all orthologous SNPs. The *ompA* genotypes are included as the first letters in the designations of the isolates. The scale bar denotes number of SNPs. The phylogeny shows the extremely close relationship of the Australian ocular (red) and UGT (blue) serovar B isolates. For the strains in purple, the records of anatomical site of isolation were unavailable.

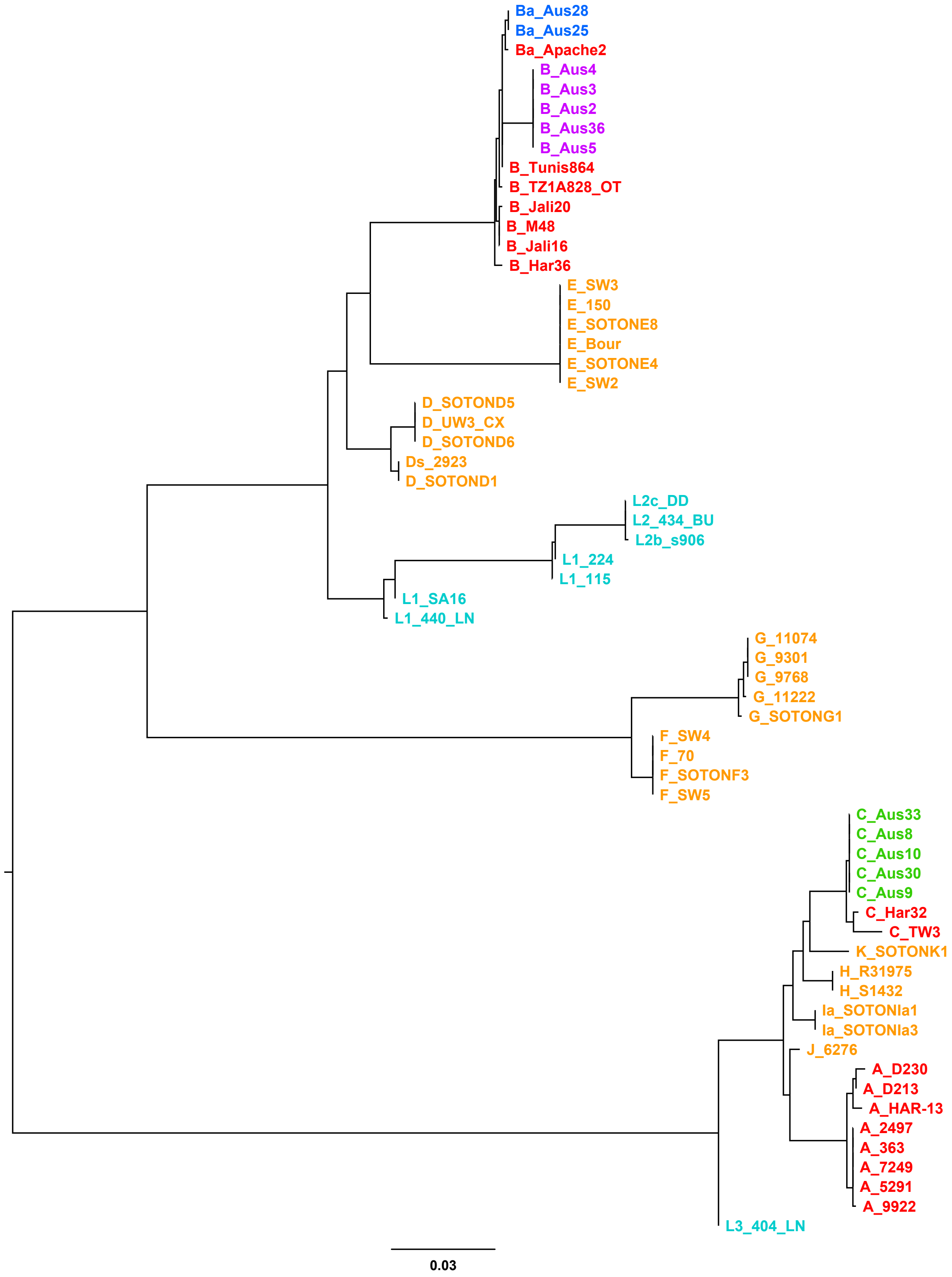

Supplementary Figure 6 | *OmpA* phylogeny. Maximum likelihood reconstruction of the phylogeny of *C. trachomatis ompA* gene sequences. The *ompA* genotypes are included as the first letters in the isolate designations. The strains are colour coded: red – Oc1 classical trachoma strains; purple – Oc2 genotype B trachoma strains; blue – Oc3 genotype Ba trachoma strains; green – Oc3 genotype C trachoma strains; orange – urogenital strains; teal – LGV strains.

Supplementary Table 1 | Outcomes of sample collection, culturing and serotyping

A. Samples collected, and success rates of culturing and serotype determination.

	Women	Children			
	Cervix	Eye	Nasopharynx	Rectum	Total
Number of specimens cultured	1219	1686	1759	379	4863
Positive cultures (%)	152 (13)	145 (9)	58 (4)	8(2)	363 (8)
Successful passage (%)	76 (50)	101 (70)	38 (66)	3 (38)	218 (60)
Serotype determined (%)	44 (58)	87 (86)	25 (66)	2 (67)	158 (72)

B. Results of serotype determination.

	Ocular serotypes				Urogenital serotypes							
	B	Ba	B/Ba#	C	B+E*	C/J#	D	D/D'#	E	F	G	K
**West Coast**												
Cervix	1						1					
Eye	20	6	3				2	1				
Nasopharynx	5	5	1				2	1				
Rectum	1											
**Tropical Coast**												
Cervix	6				1	1			8(1)	2	2	1
Eye				22					1			
Nasopharynx				4						1		
Rectum												
**Cattle Country**												
Eye	23	2		5						1		
Nasopharynx	6											
Rectum	1											
**Darwin**												
Cervix	7(5)				3(3)				3(1)	1(1)		3(3)
Eye									1			
Nasopharynx												
Rectum												
**Other areas**												
Cervix	2				1(1)					1		

#Inconsistent or ambiguous results
*Mixed strains
( ) Number of samples from non-Aboriginal participants

Supplementary Table 2 | Accession numbers for all strains in this study
For all the strains the table shows either NCBI Genbank accession numbers or NCBI Sequence Read Archive (SRA) accession numbers.

Isolate name	ERS*	ERR**	Accession number	Previously published
A_2497	-	-	FM872306	Harris et al 2012 [1]
A_363	ERS017900	ERR034213	-	Harris et al 2012 [1]
A_5291	ERS017901	ERR034214	-	Harris et al 2012 [1]
A_7249	ERS017902	ERR034215	-	Harris et al 2012 [1]
A_D213	ERS177838	ERR175652	-	This study
A_HAR13	-	-	CP000051	Carlson et al 2005 [2]
B_Aus2	ERS153019	ERR189742	-	This study
B_Aus3	ERS153020	ERR189743	-	This study
B_Aus4	ERS153021	ERR189744	-	This study
B_Aus5	ERS153022	ERR189745	-	This study
B_Aus36	ERS351385	ERR386225	-	This study
B_Aus40	ERS747489	-	-	This study
B_Aus41	ERS747490	-	-	This study
B_Aus42	ERS747491	-	-	This study
B_Aus43	ERS747492	-	-	This study
B_Aus44	ERS747493	-	-	This study
B_Aus45	ERS747494	-	-	This study
B_HAR36	ERS153013	ERR189736	-	This study
B_Jali20	-	-	FM872308	Seth-Smith et al 2009 [3]
B_TZ1A828	-	-	FM872307	Seth-Smith et al 2009 [3]
Ba_Apache2	ERS095032	ERR140762	-	This study
Ba_Aus25	ERS351392	ERR386232	-	This study
Ba_Aus28	ERS351377	ERR386222	-	This study
C_Aus10	ERS153046	ERR189769	-	This study
C_Aus30	ERS351383	ERR386223	-	This study
C_Aus33	ERS351384	ERR386224	-	This study

C_Aus8	ERS153047	ERR189770	-	This study
C_Aus9	ERS153045	ERR189768	-	This study
C_TW3	ERS177778		-	This study
C_UW1	ERS177816	ERR175630	-	This study
D_SotonD1	ERS008761	ERR027327	-	Harris et al 2012 [1]
D_SotonD5	ERS013787	ERR026547	-	Harris et al 2012 [1]
D_SotonD6	ERS008762	ERR027328	-	Harris et al 2012 [1]
D_UW3	-	-	AE001273	Stephens et al 1998 [4]
Ds_2923	-	-	ACFJ01000001	Suchland et al 2008 [5]
E_11023	-	-	CP001890	Jeffrey et al 2010
E_150	-	-	CP001886	Jeffrey et al 2010
E_Bour	ERS001401	ERR008578	-	Harris et al 2012 [1]
E_SotonE4	ERS013791	ERR026551	-	Harris et al 2012 [1]
E_SotonE8	ERS008763	ERR027329	-	Harris et al 2012 [1]
E_SW2	ERS001397	ERR008596	-	Harris et al 2012 [1]
E_SW3	ERS001406	ERR008589	-	Harris et al 2012 [1]
F_70			ABYF1000001	Jeffrey et al 2010 [6]
F_SotonF3	ERS008764	ERR027330	-	Harris et al 2012 [1]
F_SW4	ERS001414	ERR008588	-	Harris et al 2012 [1]
F_SW5	ERS001415	ERR008582	-	Harris et al 2012 [1]
G_11074	-	-	CP001889	Jeffrey et al 2010 [6]
G_11222	-	-	CP001888	Jeffrey et al 2010 [6]
G_9301	-	-	CP001930	Jeffrey et al 2010 [6]
G_9768	-	-	CP001887	Jeffrey et al 2010 [6]
G_SotonG1	ERS013800	ERR026560	-	Harris et al 2012 [1]
H_R31975	ERS082923	ERR111606	-	This study
H_S1432	ERS082985	ERR108303	-	This study
Ia_SotonIa1	ERS013804	ERR026555	-	Harris et al 2012 [1]
Ia_SotonIa3	ERS013805	ERR026565	-	Harris et al 2012 [1]

J_6276	-	-	ABYD01000001	Suchland et al 2008 [5]
K_SotonK1	ERS013799	ERR026559	-	Harris et al 2012 [1]
L1_115	ERS001411	ERR008593	-	Harris et al 2012 [1]
L1_224	ERS001404	ERR008580	-	Harris et al 2012 [1]
L1_440	ERS001396	ERR008595	-	Harris et al 2012 [1]
L1_SA16	ERS003316	ERR019529	-	Harris et al 2012 [1]
L2_434	-	-	AM884176	Thomson et al 2008 [7]
L2_LST	ERS003315	ERR019528	-	Harris et al 2012 [1]
L2b_795	ERS001409	ERR008586	-	Harris et al 2012 [1]
L2b_8200	ERS004108	ERR021952	-	Harris et al 2012 [1]
L2b_C1	ERS001398	ERR008579	-	Harris et al 2012 [1]
L2b_C2	ERS001399	ERR008592	-	Harris et al 2012 [1]
L2b_CV204	ERS003307	ERR019531	-	Harris et al 2012 [1]
L2b_UCH1	ERS001407	ERR008581	-	Harris et al 2012 [1]
L2b_UCH2	ERS001405	ERR008587	-	Harris et al 2012 [1]
L3_404	ERS001416	ERR008583	-	Harris et al 2012 [1]

* ERS = Experiment sample information accession number
** ERR = Experiment run accession number

SUPPLEMENTARY REFERENCES

1. Harris, S.R. *et al.* Whole-genome analysis of diverse Chlamydia trachomatis strains identifies phylogenetic relationships masked by current clinical typing. *Nat Genet* **44**, 413-9, S1 (2012).
2. Carlson, J.H., Porcella, S.F., McClarty, G. & Caldwell, H.D. Comparative genomic analysis of Chlamydia trachomatis oculotropic and genitotropic strains. *Infect Immun* **73**, 6407-18 (2005).
3. Seth-Smith, H.M. *et al.* Co-evolution of genomes and plasmids within Chlamydia trachomatis and the emergence in Sweden of a new variant strain. *BMC Genomics* **10**, 239 (2009).
4. Stephens, R.S. *et al.* Genome sequence of an obligate intracellular pathogen of humans: Chlamydia trachomatis. *Science* **282**, 754-9 (1998).
5. Suchland, R.J. *et al.* Identification of concomitant infection with Chlamydia trachomatis IncA-negative mutant and wild-type strains by genomic, transcriptional, and biological characterizations. *Infect Immun* **76**, 5438-46 (2008).
6. Jeffrey, B.M. *et al.* Genome sequencing of recent clinical Chlamydia trachomatis strains identifies loci associated with tissue tropism and regions of apparent recombination. *Infect Immun* **78**, 2544-53 (2010).
7. Thomson, N.R. *et al.* Chlamydia trachomatis: genome sequence analysis of lymphogranuloma venereum isolates. *Genome Res* **18**, 161-71 (2008).
